# Comparative metabolomics of leaves and stems of three Italian olive cultivars under drought stress

**DOI:** 10.3389/fpls.2024.1408731

**Published:** 2024-07-03

**Authors:** Sara Parri, Giampiero Cai, Marco Romi, Claudio Cantini, Diana C. G. A. Pinto, Artur M. S. Silva, Maria Celeste Pereira Dias

**Affiliations:** ^1^ Department of Life Sciences, University of Siena, Siena, Italy; ^2^ Institute for BioEconomy (IBE), National Research Council (CNR), Follonica, Italy; ^3^ LAQV-REQUIMTE, Department of Chemistry, University of Aveiro, Aveiro, Portugal; ^4^ Center for Functional Ecology, Department of Life Sciences, University of Coimbra, Coimbra, Portugal

**Keywords:** *Olea europaea*, drought stress, metabolomic, UHPLC-MS, GC-MS

## Abstract

The Mediterranean will be one of the focal points of climate change. The predicted dry and hot summers will lead to water scarcity in agriculture, which may limit crop production and growth. The olive tree serves as a model woody plant for studying drought stress and improving water resource management; thus, it is critical to identify genotypes that are more drought tolerant and perform better under low irrigation or even rainfed conditions. In this study, the metabolomic approach was used to highlight variations in metabolites in stems and leaves of three Italian olive cultivars (previously characterized physiologically) under two and four weeks of drought stress. Phenolic and lipophilic profiles were obtained by gas chromatography-mass spectrometry and ultra-high performance liquid chromatography-mass spectrometry, respectively. The findings identified the leaf as the primary organ in which phenolic variations occurred. The Maurino cultivar exhibited a strong stress response in the form of phenolic compound accumulation, most likely to counteract oxidative stress. The phenolic compound content of ‘Giarraffa’ and ‘Leccino’ plants remained relatively stable whether they were exposed to drought or not. Variations in the lipid profile occurred in leaves and stems of all the cultivars. A high accumulation of compounds related to epicuticular wax components was observed in the leaf of ‘Giarraffa’, while a strong reduction of lipids and long-chain alkanes occurred in ‘Maurino’ when exposed to drought stress conditions.

## Introduction

The olive tree (*Olea europaea* L.) is one of the most important crops in the Mediterranean because of the economic and cultural value of its main product, olive oil. Three Mediterranean countries (Spain, Italy, and Greece), along with Portugal, produce 99% of the EU’s olive oil, which is the largest contribution in the world, accounting for 69% of global production (European Commission, 2020). The olive tree is evolutionarily adapted to the typical Mediterranean semi-arid climate, characterized by hot and dry summers with high solar radiation and cold and wet winters. Although olive trees are traditionally rain-fed, an increasing number of olive groves are being irrigated (Carr, 2013). Irrigation, especially during the flowering period, contributes to a consistent production of olives, which is an economic advantage for the farmers. The need for irrigation has become even more urgent in the last decade, as rising temperatures have led to changes in rainfall patterns. According to the IPCC, (2023), the Mediterranean region is a climate change hotspot, with an increase in heat extremes and droughts. Due to erratic rainfall and increasing droughts, coupled with limited water availability for agricultural use, crop irrigation will compete with other sectors such as industry and human consumption (Elliott et al., 2014). One of the main strategies for addressing water scarcity in agriculture is to improve the sustainability of irrigation practices and implement new farming methods. In addition, research into locally adapted varieties and characterization of existing crop varieties is helping to develop and promote the use of more efficient water-saving varieties (Malhi et al., 2021).

Plants, including olive trees, can respond to drought or water shortage in a variety of ways. The most relevant (and even studied) responses to drought are physiological adaptations. These include the regulation of gas exchange and its effect on leaf water content and potential (Guerfel et al., 2009). In addition, olive cultivars have been found to exhibit adaptive responses, such as stomatal closure, to conserve water during drought (Torres-Ruiz et al., 2013; Haworth et al., 2018; Rodriguez-Dominguez and Brodribb, 2020). These and other physiological mechanisms play a critical role in the survival and productivity of olive trees in arid environments. The olive tree is an anisohydric species because it can reach very low water potentials and maintain basal rates of stomatal conductance even at very low soil water contents (Rossi et al., 2013). This ability allows olive trees to continue photosynthesizing and growing even in extremely dry conditions, contributing to the resilience of olive trees in arid environments (Sofo et al., 2008). While physiological responses are a relevant mechanism to counteract drought, olive plants can also use additional mechanisms such as osmotic adaptation (i.e. increase in proline), anatomical changes (i.e. thick cuticular wax on leaves or different vascular bundle diameters) and an efficient antioxidant system to face the effects of drought stress (Bacelar et al., 2006, 2007; Ahmadipour et al., 2018; Rico et al., 2023). These adaptations cooperate to help olive trees maintain their water balance and protect themselves from drought-induced oxidative stress.

Together with the mechanisms listed above responses against drought include also metabolic adaptations. Sugar metabolism is known to be involved in the response and adaptation of olive trees to drought, including changes in the levels of simple sugars such as sucrose and glucose (Bacelar et al., 2007; Brito et al., 2019; Tsamir-Rimon et al., 2021). Drought stress promotes the accumulation of monosaccharides and disaccharides, which play a critical role in drought tolerance; however, sucrose can also produce trehalose-6-phosphate and raffinose, which are equally important (Kumar et al., 2021). In addition to simple or complex carbohydrates, sugar alcohols such as mannitol and sorbitol (an isomer of mannitol) also accumulate in response to drought because of their role in osmotic adjustment (Mechri et al., 2015, 2020a). Thus, in addition to being an important source of energy in plants, carbohydrates can also be used for survival during stress conditions or for physiological recovery during rehydration (Dias et al., 2021). In addition to primary metabolism, secondary metabolism helps olive trees adapt to stress conditions. Secondary metabolites are not directly involved in plant growth and development, but they serve various functions in their interactions with the environment and include terpenoids, phenolics and alkaloids. Secondary metabolites act as scavengers of reactive oxygen species (ROS) to protect plants from lipid peroxidation and oxidative damage under stress. Furthermore, secondary metabolites could systemically alert plant tissues to implement drought stress defense processes (Yadav et al., 2021). A growing body of literature describes the metabolomic approaches that have already been used to identify the primary stress-related metabolites in olive trees and to investigate the most sensitive pathways modulated by drought. Among the phenolic compounds, luteolin-7-*O*-glucoside, quercetin-7-*O*-rutinoside, apigenin-7-*O*-glucoside, chrysoeriol-7-*O*-glucoside and oleuropein were found to accumulate in olive leaves, probably to counteract the increase in ROS formation induced by stress (Petridis et al., 2012; Mechri et al., 2020b; Dias et al., 2021). Among the lipophilic terpenoid compounds, lupeol has also been assigned an antioxidant role (Araújo et al., 2021; Dias et al., 2021). Higher levels of terpenes and long-chain alkanes were associated with leaf cuticle thickness in drought-stressed olive plants (Piccini et al., 2022), while increases in saturated/unsaturated fatty acid ratio and sterol content were associated with low membrane permeability (Dias et al., 2021). Most studies in the literature on the metabolomics of drought-stressed olive plants concern the leaf system, but there are few studies on the variation of the content of lipophilic and phenolic compounds in drought-stressed olive stems (Mechri et al., 2020a). Jiménez-Herrera et al. (2019) found that the total content of triterpenes and the phenolic compounds oleuropein, hydroxytyrosol, and tyrosol were lower in stems than in leaves; they also reported a decrease in hydroxytyrosol, tyrosol, and total triterpenes and an increase in oleuropein in response to drought. While leaves are often the focus of drought stress studies, the role of the stem in coping with drought stress is also important, as it acts as a water reservoir and water distributor, reducing the damage caused by water shortages (Traversari et al., 2018). The study of the metabolic behavior of both leaves and stems in drought-stressed plants aims to unravel the mechanisms that different olive cultivars individually implement by allocating specific secondary metabolites between the two organs.

In a previous work (Parri et al., 2023), the drought response of three Italian olive cultivars (‘Giarraffa’, ‘Leccino’ and ‘Maurino’) was studied and it was found that ‘Giarraffa’ decreased its stomatal conductance (*g_s_*) very early after the onset of stress, while ‘Maurino’ did so later; ‘Leccino’ showed an intermediate response. Interestingly, these *g_s_* differences did not significantly affect leaf water content but resulted in higher stem water content in ‘Giarraffa’. The cultivars also showed different levels of electrolyte leakage (EL) under drought stress, with ‘Maurino’ having the highest and ‘Giarraffa’ the lowest, probably due to its earlier *g_s_* reduction. We hypothesize that ‘Maurino’ and ‘Leccino’ may have used metabolic changes to signal drought and manage ROS generation and oxidative stress. In this manuscript, we analyzed the metabolic changes that occurred in Leccino, Maurino, and Giarraffa cultivars, both in control plants and in plants exposed to two and four weeks of drought stress, using gas chromatography-mass spectrometry (GC-MS)-based profiling of lipophilic metabolites and ultra-high performance liquid chromatography-mass spectrometry (UHPLC-MS)-based profiling of phenolic metabolites. The analysis was carried out on both leaves and stems in order to reveal metabolic differences that could explain the different physiological behavior of the three cultivars.

## Materials and methods

### Plant growth conditions and drought stress treatment

Olive plants were grown and drought-stressed as previously described (Parri et al., 2023). Briefly, 18-month-old olive trees (*Olea europaea* L., cultivars Leccino, Maurino, and Giarraffa) were grown in 4-L pots (15x15x20 cm) with a substrate of 50% peat and 50% pumice (Tosca et al., 2019). The plants were then transferred to a growth chamber with LED illumination for flowering and growth (photoperiod of 12 hours light and 12 hours dark), which provided a photosynthetic photon flux of 450-550 µmol m^-2^ s^-1^ at plants’ leaves level. After one week of acclimation, plants of each cultivar were divided into two groups: a fully irrigated control group (CTRL) and a drought-stressed group (DS) that was water deprived for 4 weeks. The water deficit period was divided into 3 time points (t0, t2, t4) corresponding to the onset of withholding irrigation, the second and the fourth weeks of water deprivation, respectively. Three plants per cultivar were sacrificed for stem analysis at t0. At both time points, t2 and t4, three plants from each of the drought-stressed and control groups were sacrificed for stem analysis. Leaf samples were taken from at least 6 individuals at all time points. Experimental design is shown in **Supplementary Figure S1** of the Supplementary Material. Temperature and humidity were recorded hourly (average temperature of 27.5°C and humidity of 51.1%). The pots inside the chamber were rotated weekly to avoid any positional effects.

The growth of the plants was monitored by measuring the difference between the height at t4 and at t0 of 6 plants for each experimental group. The control group of ‘Giarraffa’, ‘Leccino’ and ‘Maurino’ grew by about 2.6, 2.4, 4.3 cm respectively, while the drought stressed groups of ‘Giarraffa’, ‘Leccino’ and ‘Maurino’ had a growth of about 2.2, 1.7, 1.8 cm.

### Phenolic and lipophilic compounds extraction

Frozen olive leaves and stems harvested at t0, t2 and t4 from both control and stressed groups of cultivars ‘Giarraffa’, ‘Leccino’ and ‘Maurino’ were dried at 40°C for 7 days. The dried plant material was then finely ground in a mill to obtain a powder for metabolite extraction. The ground material was combined with n-hexane (1:10 w:v) at room temperature with magnetic stirring for 48 hours. The n-hexane was then decanted and a second extraction was performed with fresh n-hexane for a further 24 hours. The combined n-hexane extracts were concentrated to dryness in a rotary evaporator under reduced pressure to give the crude extracts, which were air dried for one week. The pellet resulting from the n-hexane extraction was further air dried and then subjected to extraction with 50 mL of methanol to isolate phenolic compounds. The methanol extraction involved two cycles: a first cycle of 48 hours at room temperature with magnetic stirring, followed by removal of the methanol, and a second cycle of 24 hours with fresh methanol. The methanol extracts from both cycles were pooled and concentrated to dryness in a rotary evaporator under reduced pressure. Finally, the concentrated extract was air dried for two weeks.

### GC-MS analysis

The extracted samples from the n-hexane extraction were weighed and prepared for silylation. In a glass tube, a 200 μL aliquot of the extract was mixed with 200 μL of tetracosane solution (0.5 mg mL^-1^), 250 μL of pyridine, 250 μL of N,O-bis(trimethylsilyl)trifluoroacetamide and 50 μL of trimethylsilyl chloride. The mixture was then incubated at 70°C for 40 minutes. After the incubation period, 1 μL of the silylated extract was injected into a gas chromatography-mass spectrometry (GC-MS) apparatus (QP2010 Ultra Shimadzu, Kyoto, Japan). The chromatography conditions followed the protocols described in Dias et al. Dias et al. (2019). Data were collected at a rate of one scan s^−1^ over a range of *m/z* 33–750, as described in Dias et al. (2019). For identification of lipophilic compounds, chromatographic peaks were compared with entries in mass spectral databases such as the NIST14 Mass Spectral Library and the Wiley Registry^®^ of Mass Spectral Data. In addition, comparison was made with mass spectra and retention times of pure compounds prepared and analyzed under conditions similar to those of the samples. The identification of some compounds was done using the retention index relative to n-alkanes injected in the same chromatographic conditions as described in Costa et al. (2021). Calibration curves were established for quantification purposes using standard compounds representing the major families of compounds present in the extracts. These standards included maltose for sugars (y = 0. 0416x + 0.0117, *R^2^* = 0.99), palmitic acid for fatty acids (y = 0. 0941x + 0.5236, *R^2^* = 0.99), octadecane for alkanes (y = 0. 0942x + 0.061, *R^2^* = 0.99), octadecanol for alcohols (y = 0. 2322x + 0.0474, *R^2^* = 0.99), and cholesterol for sterols and terpenes (y = 0. 0596x + 0.0447, *R^2^* = 0.99). The detection and quantification limits (LOD and LOQ, respectively) were determined from the parameters of the calibration curves. Quantitative results were expressed as grams per kilogram dry weight (g/Kg DW) and are presented as the mean ± standard deviation of three independent analyses.

### UHPLC-MS analysis

Methanol residues were weighed and resuspended in methanol to achieve a final concentration of 10 mg/mL. Next, 4 μL of the solution was injected into a Thermo Scientific Ultimate 3000RSLC Dionex (Waltham, MA, USA) equipped with a Dionex UltiMate 3000 RS diode array detector coupled with a mass spectrometer operating in negative ion mode. The analysis was performed using a Hypersil GOLD column (1.9 μm particle diameter, Thermo Scientific, Lenexa, KS, USA) as described in Dias et al. (2019). A mass range of 50.00–2000.00 *m/z* was covered. Compound identification was also performed as described in the same reference. UV-Vis spectral data were collected between 250 and 500 nm, and the chromatogram profile was recorded at 280 nm. A semi-quantitative analysis was performed using peak integration through the standard external method. The peaks were identified by comparing the retention times, UV-Vis spectra, and spectral data obtained from the reference compounds. The calibration curves were calculated using the reference compounds (quercetin for flavonoids and oleuropein for secoiridoids) to determine the detection and quantification limits. The calibration curves were generated by injecting various concentrations of quercetin and oleuropein. The equation for quercetin was y = 4 × 106x − 390882 with an *R^2^* value of 0.99, where x represents the amount of the compound in mg/mL and y represents the peak area obtained in the chromatogram. The equation for oleuropein was y = 106x − 6948 with an *R^2^* value of 0.98, where x represents the amount of the compound in mg/mL and y represents the peak area obtained in the chromatogram. The detection and quantification limits (LOD and LOQ, respectively) were determined from the parameters of the calibration curves. The same conditions were used for the sample analysis. The compound concentration was measured in milligrams per gram of tissue dry weight. The mean ± standard deviation of three independent analyses per sampling time and treatment are presented.

### Statistical analysis

A “group” is defined by the cultivar considered (Giarraffa, Leccino and Maurino), the irrigation treatment (control, CTRL or drought stressed, DS) and the time of sampling (beginning of stress, t0; two weeks after the beginning, t2; four weeks after the beginning, t4). Each group was analyzed in triplicate. Data distributions were tested for normality using the Shapiro-Wilk normality test in R studio (ver. 4.2.2, R core team, Vienna, Austria, 2022). Repeated measures ANOVA was used to test the significance of each of the three factors (treatment, cultivar and organ) and their interactions. *Post hoc* This statistical analysis was conducted using the Systat 11 statistical package (Systat Software Inc., Richmond, CA, USA). Due to the absence of the “MAU DS t4” group of stems, leaf and stem data were processed separately for the following analysis. This allowed not to exclude this group from the leaf data processing. For the following analyses, first, metabolite contents were normalized by Z-score and missing data were replaced by a value of 0.0001. Hierarchical clustering analysis (HCA) was performed on metabolites using Euclidean distance as the similarity metric and the complete linkage method between groups. The resulting heat maps and clustering were generated using Rstudio (ver. 4.2.2, R core team, Vienna, Austria, 2022) with the package “pheatmap” version 1.0.12. The observation inputs correspond to the variance of each group at t2 and t4, when the drought stress occurred. The variables input were the classes of both phenolic and lipophilic compounds. The Principal Component Analysis (PCA) biplots were realized with Rstudio (ver. 4.2.2, R core team, Vienna, Austria, 2022) with the package “Factoextra” version 1.0.7.

## Results

### Profile of phenolic secondary metabolites

As a first general observation, 20 phenolic compounds were identified (16 flavonoids and 4 secoiridoids): 6 in both plant organs, 3 in stems, and 10 in leaves only. Retention times, *m/z*, MS2 (*m/z*) fragments and mean ± standard deviations for each experimental group at t0 and t2 are summarized in **Supplementary Tables S1**, **S2** for leaves, and in **Supplementary Tables S3**–**S5** for stems (**Supplementary Material**). As part of an ongoing collaboration, data on phenolic compounds from leaves at t4 are published in Cerri et al. (2024) (**Table 1**); in that manuscript, olive leaf extracts were characterized and tested on human umbilical vein endothelial cells (HUVECs). **Tables 1**–**3** show the repeated measures ANOVA results for phenolic compounds found in the stem and leaf, leaf only, and stem only, respectively. Results indicate that cultivar had a significant effect on all compounds examined (*p-*value < 0.05). Indeed, the cultivars studied contained significantly different amounts of the identified compounds, with some compounds found only in one cultivar (for example, fraxamoside was found only in the stem of ‘Leccino’). Unlike the other two cultivars, in the leaf of ‘Giarraffa’ luteolin-7-*O*-rutinoside was not detected. Instead, another isomer 1 of luteolin-7-*O*-glucoside was detected in this cultivar, but not in ‘Leccino’ and ‘Maurino’. In terms of leaves, chrysoeriol-7-*O*-glucoside and luteolin-7-*O*-glucoside is.3 were found only in the cultivar Leccino. The “organ” parameter significantly influenced the amount of compounds found in both leaves and stems. For example, luteolin, apigenin, and oleuropein were more abundant in leaves, while luteolin-7-*O*-glucoside and dihydroquercetin were more abundant in the stems.

**Table 1:** Repeated measures ANOVA table carried out with the contents (mg/g DW) of phenolic compounds found in both stem and leaf of three olive cultivars (Giarraffa, Leccino and Maurino) irrigated (CTRL) or exposed to drought (DS).

Compound	dihydroquercetin	luteolin-7-*O*-glucoside	oleuropein	chrysoeriol-7-*O*-glucoside	luteolin	apigenin
Cultivar (C)						
**Leccino**	3.733	3.647	1.451	0.576	3.633	3.427
**Maurino**	3.049	3.641	4.478	1.953	2.653	1.730
**Giarraffa**	2.817	6.847	0.944	1.555	1.971	2.243
**p-value**	< 0.001	< 0.001	< 0.001	< 0.001	< 0.001	< 0.001
Organ (O)						
**Leaf**	2.942	3.871	2.981	1.729	4.474	3.626
**Stem**	3.457	5.552	1.602	0.994	1.031	1.307
**p-value**	0.005	< 0.001	< 0.001	< 0.001	< 0.001	< 0.001
Treatment (T)						
**Control**	3.182	4.437	1.35	1.312	2.965	2.582
**Stressed**	3.218	4.986	3.232	1.411	2.539	2.351
**p-value**	0.832	0.046	< 0.001	0.002	< 0.001	< 0.001
Time						
**t0**	3.416	4.686	1.621	1.369	3.044	2.661
**t2**	3.322	4.616	1.796	1.437	2.715	2.535
**t4**	2.861	4.833	3.456	1.279	2.497	2.204
T x C						
**p-value**	0.028	0.552	< 0.001	< 0.001	< 0.001	< 0.001
T x O						
**p-value**	0.304	0.406	< 0.001	0.112	< 0.001	< 0.001
C x O						
**p-value**	< 0.001	< 0.001	< 0.001	< 0.001	< 0.001	< 0.001
T x C x O						
**p-value**	0.716	0.096	< 0.001	< 0.001	< 0.001	< 0.001

Samples were taken at the beginning of the drought stress (t0) and two (t2) and four (t4) weeks after the start of water withholding. Each value represents the mean.

**Table 2:** Repeated measures ANOVA table carried out with the contents (mg/g DW) of phenolic compounds found in the leaves of three olive cultivars (Giarraffa, Leccino and Maurino) irrigated (CTRL) or exposed to drought (DS).

Compound	oleuropein aglicone	aldehydic form of decarboxyl elenolic acid	luteolin-7-*O*-rutinoside	luteolin-7-*O*-glucoside is.1	apigenin-*O*-dideoxyhexoside-hexoxide	apigenin-7-*O*-rutinoside is.1	apigenin-7-*O*-rutinoside is.2	luteolin-7-*O*-glucoside is.3	apigenin-7-*O*-rutinoside is.3	diosmetin
Cultivar (C)										
**Leccino**	0.207	3.205	2.822	0	2.791	5.03	3.074	0	2.154	2.765
**Maurino**	1.221	0.684	4.217	0	2.243	2.869	2.396	2.600	2.483	3.117
**Giarraffa**	0.937	0.512	0	2.104	1.809	3.147	2.359	2.075	1.907	2.469
***p*-value**	< 0.001	< 0.001	< 0.001	< 0.001	0.019	< 0.001	< 0.001	< 0.001	0.006	< 0.001
Treatment (T)										
**Control**	0.674	1.821	1.857	0.669	2.26	3.675	2.558	1.557	2.135	2.991
**Stressed**	0.930	1.113	2.836	0.734	2.302	3.689	2.662	1.560	2.228	2.576
***p*-value**	< 0.001	0.046	< 0.001	< 0.001	0.008	0.594	< 0.001	0.912	0.449	< 0.001
Time										
**t0**	0.639	1.823	1.852	0.667	2.273	3.687	2.569	1.594	2.132	2.98
**t2**	0.785	1.266	1.817	0.716	2.253	3.644	2.594	1.596	2.253	2.657
**t4**	0.942	1.313	3.370	0.722	2.317	3.715	2.665	1.557	2.159	2.714
C x T										
***p*-value**	< 0.001	< 0.001	< 0.001	< 0.001	< 0.001	< 0.001	< 0.001	< 0.001	*0.054*	< 0.001

Samples were taken at the beginning of the drought stress (t0) and two (t2) and four (t4) weeks after the start of water withholding. Each value represents the mean.

**Table 3:** Repeated measures ANOVA table carried out with the contents (mg/g DW) of phenolic compounds found in the stems of three olive cultivars (Giarraffa, Leccino and Maurino) irrigated (CTRL) or exposed to drought (DS).

Compound	quercetin-3-*O*-glucoside	fraxamoside	quercetin
Cultivar (C)			
**Leccino**	2.33	2.249	1.126
**Maurino**	1.459	0	0.746
**Giarraffa**	2.324	0	0.781
***p*-value**	< 0.001	< 0.001	< 0.001
Treatment (T)			
**Control**	2.021	0.812	0.878
**Stressed**	2.055	0.687	0.891
***p*-value**	0.819	0.692	0.679
Time			
**t0**	2.303	0.853	1.024
**t2**	2.276	0.868	1.015
**t4**	1.535	0.528	0.613
C x T			
***p*-value**	0.761	< 0.001	0.006

Samples were taken at the beginning of the drought stress (t0) and two (t2) and four (t4) weeks after the start of water withholding. Each value represents the mean.

It is noteworthy that drought treatment affected most of the compounds found in the leaves (such as oleuropein aglycone, aldehyde form of decarboxyl elenolic acid, luteolin-7-*O*-rutinoside) as well as the ubiquitous compounds (luteolin-7-*O*-glucoside, oleuropein, luteolin, and apigenin), but not the compounds found only in the stems. However, for dihydroquercetin, quercetin, apigenin-7-*O*-rutinoside is.1, and luteolin-7-*O*-glucoside is.3, which were unaffected by drought stress alone, the interaction between cultivar and treatment was significant. Only luteolin-7-*O*-glucoside was significantly affected by treatment, with no differences in cultivar-organ interactions with treatment.

### Lipophilic compounds metabolite profiles

A total of 30 lipophilic compounds were detected, belonging to sterols and terpenes, sugars, alcohols, fatty acids, and alkanes. Leaves and stems had distinct profiles, with 12 compounds found in both leaves and stems, 12 in leaves and 6 in stems only. Retention times and mean ± standard deviations for each experimental group at t0, t2 and t4 are summarized in **Supplementary Tables S6**–**S8** for leaves and in **Supplementary Tables S9**–**S11** for stems (**Supplementary Material**). **Tables 4**–**6** show the results of the repeated measures ANOVA for lipophilic compounds found in both stem and leaf, leaf only, and stem only, respectively. Cultivar had a significant effect on all identified compounds except for sugar turanose in stems. Similarly, alpha-tocopherol was not detected in the leaves of ‘Maurino’, and phytol was only found in the leaves of ‘Giarraffa’. The “organ” parameter significantly influenced the compound profile of both stems and leaves. In fact, the leaves contained more alpha-D-mannopyranose, alpha-D-sorbitol, alpha-D-glucose, palmitic acid, stearic acid, alpha-monopalmitin, alpha-monopalmitin derivatives, lupeol derivatives, and ursolic aldehyde than the stems. Only oleic acid and alpha-linolenic acid were more abundant in the stems. Ursolic acid was the only compound for which neither organ, treatment, or interaction had a significant effect. Other compounds not significantly affected by drought stress included alpha- and beta-amyrin, two long-chain alkanes in leaves, and turanose in stems. However, treatment, cultivar, and organ interactions lose significance for D-glucose, whereas drought becomes significant for alpha-amyrin and the second long chain alkane found in the cultivar by treatment interaction.

**Table 4:** Repeated measures ANOVA table carried out with the contents (mg/g DW) of lipophilic compounds found in both stem and leaf of three olive cultivars (Giarraffa, Leccino and Maurino) irrigated (CTRL) or exposed to drought (DS).

Compound	α-D-mannopyranose	D-glucose	D-sorbitol	palmitic acid	α-linolenic acid	oleic acid	stearic acid	α-monopalmitin	α-monopalmitin derivative	lupeol derivative	ursolic acid	ursolic acid aldeyde
Cultivar (C)												
**Leccino**	0.132	0.178	0.873	7.107	2.626	6.621	6.732	6.251	6.640	0.516	0.922	0.507
**Maurino**	0.131	0.140	0.635	6.272	1.629	4.871	5.638	5.131	5.755	0.568	0.672	0.517
**Giarraffa**	0.068	0.157	0.82	8.956	7.365	2.978	8.644	8.242	8.627	1.132	1.186	0.305
***p*-value**	< 0.001	< 0.001	< 0.001	< 0.001	< 0.001	< 0.001	< 0.001	< 0.001	< 0.001	< 0.001	0.010	< 0.001
Organ (O)												
**Leaf**	0.126	0.205	0.963	9.357	2.937	4.794	8.759	8.215	8.330	1.051	1.319	0.5
**Stem**	0.095	0.112	0.588	5.534	4.809	4.852	5.250	4.867	5.685	0.426	0.534	0.387
***p*-value**	< 0.001	0.024	< 0.001	0.003	< 0.001	0.001	< 0.001	< 0.001	< 0.001	< 0.001	0.219	< 0.001
Treatment (T)												
**Control**	0.115	0.179	0.792	7.299	3.335	4.771	6.906	6.404	6.917	0.831	0.911	0.425
**Stressed**	0.111	0.138	0.757	7.591	4.412	4.875	7.103	6.679	7.098	0.646	0.942	0.462
***p*-value**	< 0.001	0.004	0.012	< 0.001	< 0.001	< 0.001	< 0.001	< 0.001	< 0.001	< 0.001	0.612	< 0.001
Time												
**t0**	0.115	0.163	0.763	7.194	4.011	4.625	6.744	6.183	6.815	0.825	0.890	0.451
**t2**	0.111	0.147	0.690	7.844	4.411	5.139	7.217	6.721	7.208	0.850	0.854	0.508
**t4**	0.106	0.166	0.873	7.298	3.197	4.705	7.053	6.720	6.999	0.541	1.036	0.372
T x C												
***p*-value**	< 0.001	0.063	< 0.001	< 0.001	< 0.001	< 0.001	< 0.001	< 0.001	< 0.001	< 0.001	0.578	< 0.001
T x O												
***p*-value**	< 0.001	0.063	< 0.001	< 0.001	< 0.001	< 0.001	< 0.001	< 0.001	< 0.001	< 0.001	0.907	< 0.001
C x O												
***p*-value**	< 0.001	0.082	< 0.001	< 0.001	< 0.001	< 0.001	< 0.001	< 0.001	< 0.001	< 0.001	0.779	< 0.001
T x C x O												
***p*-value**	< 0.001	*0.212*	< 0.001	< 0.001	< 0.001	< 0.001	< 0.001	< 0.001	< 0.001	< 0.001	0.170	< 0.001

Samples were taken at the beginning of the drought stress (t0) and two (t2) and four (t4) weeks after the start of water withholding. Each value represents the mean.

**Table 5:** Repeated measures ANOVA table carried out with the contents (mg/g DW) of lipophilic compounds found in the leaves of three olive cultivars (Giarraffa, Leccino and Maurino) irrigated (CTRL) or exposed to drought (DS).

Compound	linoleic acid	oleic acid derivative	neophytadiene	phytol	squalene	α-amyrin	β-amyrin	alpha tocopherol	LCAlkane 1	LCAlkane 2	LCAlkane 3	LCAlkane 4
Cultivar (C)												
**Leccino**	7.161	7.193	0.536	0.515	0.694	0.727	0.755	0.752	0.976	1.289	1.809	1.202
**Maurino**	6.851	6.850	0.690	0	0.651	0.731	0.750	0	1.095	1.443	1.970	1.144
**Giarraffa**	3.569	0.000	0.654	0	0.000	1.129	1.262	0.224	1.614	2.202	2.926	2.057
***p*-value**	< 0.001	< 0.001	< 0.001	< 0.001	< 0.001	< 0.001	< 0.001	< 0.001	< 0.001	< 0.001	< 0.001	< 0.001
Treatment (T)												
**Control**	6.064	4.860	0.814	0.215	0.460	0.814	0.959	0.240	1.264	1.686	2.340	1.501
**Stressed**	5.656	4.502	0.440	0.129	0.437	0.911	0.886	0.410	1.193	1.603	2.129	1.434
***p*-value**	< 0.001	< 0.001	< 0.001	0.008	0.009	0.153	0.052	< 0.001	0.029	0.207	0.122	0.399
Time												
**t0**	4.297	4.308	0.740	0.196	0.405	0.814	0.914	0.224	1.207	1.615	2.282	1.410
**t2**	4.557	4.573	0.600	0.193	0.433	0.919	0.922	0.235	1.229	1.653	2.253	1.435
**t4**	8.727	5.162	0.540	0.125	0.508	0.753	0.931	0.517	1.250	1.665	2.170	1.557
C x T												
***p*-value**	< 0.001	< 0.001	< 0.001	*0.019*	< 0.001	0.031	0.133	< 0.001	0.007	0.042	0.144	0.176

Samples were taken at the beginning of the drought stress (t0) and two (t2) and four (t4) weeks after the start of water withholding. Each value represents the mean.

**Table 6:** Repeated measures ANOVA table carried out with the contents (mg/g DW) of lipophilic compounds found in the stems of three olive cultivars (Giarraffa, Leccino and Maurino) irrigated (CTRL) or exposed to drought (DS).

Compound	pentadecan-1-ol derivative	palmitic acid derivative	palmitic acid derivative	turanose	monostearin	stigmast-5-ene
Cultivar (C)						
**Leccino**	0.398	5.484	5.271	0.078	6.026	0.777
**Maurino**	0.292	3.478	3.318	0.137	4.478	0.520
**Giarraffa**	0.554	6.083	5.982	0.14	6.634	0.748
***p*-value**	< 0.001	< 0.001	< 0.001	0.252	< 0.001	< 0.001
Treatment (T)						
**Control**	0.330	4.554	4.384	0.320	5.334	0.652
**Stressed**	0.499	5.479	5.330	0.105	6.092	0.711
***p*-value**	< 0.001	< 0.001	< 0.001	0.418	< 0.001	< 0.001
Time						
**t0**	0.436	4.795	4.573	0.174	5.789	0.726
**t2**	0.504	5.525	5.331	0.104	6.233	0.761
**t4**	0.304	4.73	4.667	0.078	5.116	0.558
C x T						
***p*-value**	< 0.001	< 0.001	< 0.001	0.320	< 0.001	< 0.001

Samples were taken at the beginning of the drought stress (t0) and two (t2) and four (t4) weeks after the start of water withholding. Each value represents the mean.

### HCA of leaf metabolites

**Figure 1:**
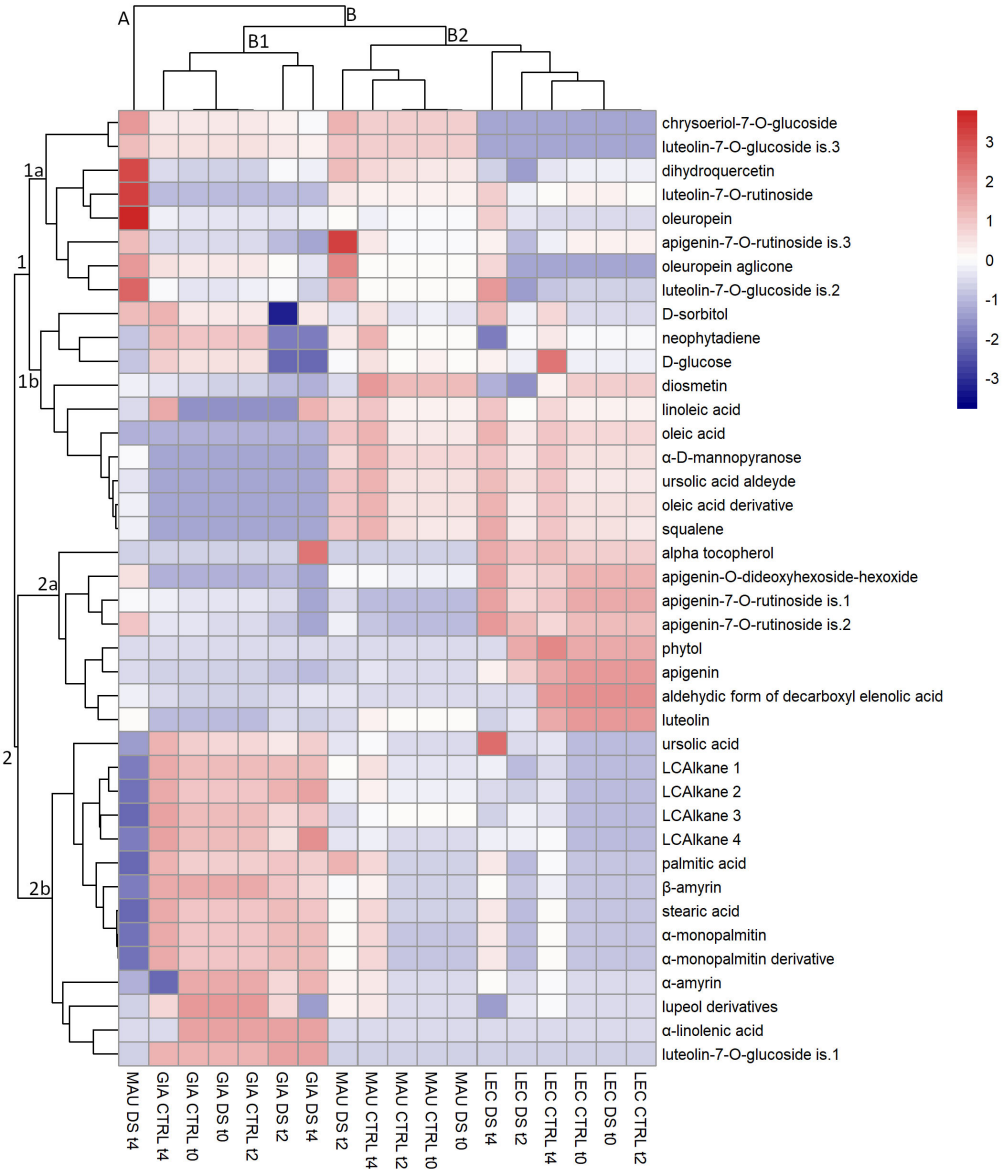
Heat map of metabolites extracted from the leaves of olive cultivars Giarraffa (GIA), Leccino (LEC) and Maurino (MAU) irrigated (CTRL) or exposed to drought (DS). Samples were taken at the beginning of the drought stress (t0) and two (t2) and four (t4) weeks after the start of water deprivation. Hierarchical clustering is shown for both metabolites (left) and experimental groups (top). The red and blue colors correspond to higher and lower relative metabolite amounts normalized according to *Z*-score.

HCA analysis of leaf metabolites (**Figure 1**) revealed two major clusters (clusters 1 and 2), each with two subclusters (1a, 1b, 2a, 2b). The sub-cluster 1a consisted of 8 phenolic compounds that were very abundant in the cultivar Maurino; in particular, the metabolites luteolin-7-*O*-rutinoside, dihydroquercetin and oleuropein were very abundant in “MAU DS t4”, whereas apigenin-7-*O*-rutinoside is.3 was highly enriched in “MAU DS t2”. The metabolites of this cluster (especially chrysoeriol-7-*O*-glucoside and luteolin-7-*O*-glucoside is.3) were significantly less abundant in ‘Leccino’ regardless of time point or treatment. Furthermore, metabolites accumulated in “MAU DS t4” (including apigenin-7-*O*-rutinoside is.3) were less abundant in all ‘Giarraffa’ groups compared to the other cultivars. The sub-cluster 1b contained 10 metabolites (mainly lipophilic compounds except for the flavonoid diosmetin) the content of which was significantly lower in ‘Giarraffa’ and in “MAU DS t4” regardless of time point or treatment. “GIA DS t2” had particularly a very low content of D-glucose and D-sorbitol. Metabolites from this cluster accumulated preferentially in all other groups of ‘Leccino’ and ‘Maurino’ (except for “MAU DS t4”).

The sub-cluster 2a contains 6 phenolic compounds but only 2 lipophilic compounds. The metabolites of this sub-cluster were mainly accumulated in the cultivar ‘Leccino’ regardless of the time point and treatment, although ‘GIA DS t2’ had a high amount of alpha-tocopherol. Conversely, the compounds in this subcluster showed a lower content in both ‘Giarraffa’ and ‘Maurino’. The 14 compounds on the left in sub-cluster 2b were all lipophilic, with the exception of the flavonoid luteolin-7-*O*-glucoside is.1. The metabolites of this cluster accumulated more in ‘Giarraffa’ (regardless of time point or treatment) than in the other two cultivars; in fact, their content was particularly low in “MAU DS t4”. The HCA revealed a clear separation of leaf lipophilic and phenolic compounds, which were mostly grouped into distinct clusters.

Cultivar clustering analysis (including time points and treatments) revealed two major sub-clusters (B1 and B2) of cluster B, as well as an orphan group containing only “MAU DS t4” (cluster A), which behaved very differently from the other groups. In particular, it showed a very low content of all long-chain alkanes and other metabolites belonging to the sterol and terpene class, whereas a huge amount of many phenolic compounds of sub-cluster 1a were found, especially oleuropein, dihydroquercetin and luteolin-7-*O*-rutinoside. Sub-cluster B1 included the Giarraffa cultivar, which differed from the other two cultivars in the higher content of long-chain alkanes, sterols, and terpenes and the lower amount of some fatty acids such as oleic and linoleic acid. Within sub-cluster B1, the metabolic composition of the ‘Giarraffa’ control differed from the metabolic profile of ‘Giarraffa’ under drought stress at t2 and t4, especially for the lower amount of D-glucose, neophytadiene and D-sorbitol. Sub-cluster B2 included both ‘Maurino’ and ‘Leccino’, which behaved much more similarly to each other than to ‘Giarraffa’, especially in terms of lipophilic compounds (sub-clusters 1b and 2b). The ‘Maurino’ control and drought-stressed samples at t0 were further separated from the drought-stressed ‘Maurino’’ at t2, where there was a higher amount of apigenin-7-*O*-rutinoside is.3, oleuropein aglicone and luteolin-7-*O*-glucoside is.2. Similarly, in cultivar Leccino, the control and stressed samples at t0 differed significantly from the drought-stressed samples at t2 and t4, which contained lower levels of diosmetin and dihydroquercetin. As a result, the groups in cluster B were more similar within the cultivar than when treatment conditions were considered. In particular, sub-cluster B1 distinguished ‘Giarraffa’ from the other two cultivars. Within each cultivar cluster, the control groups formed a separate cluster from the stressed groups.

### HCA of stem metabolites

**Figure 2:**
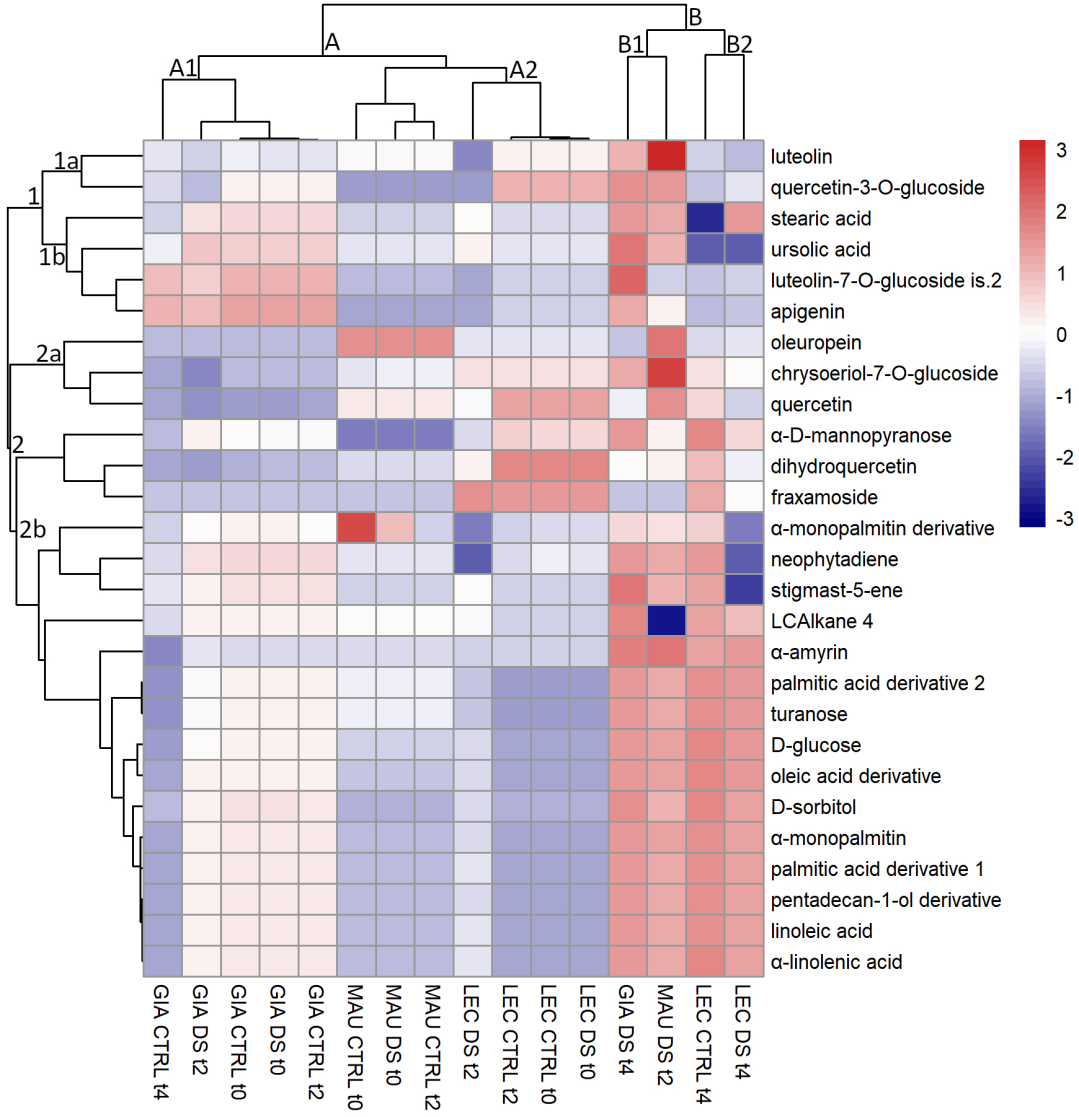
Heat map of metabolites extracted from the stems of olive cultivars Giarraffa (GIA), Leccino (LEC) and Maurino (MAU) irrigated (CTRL) or exposed to drought (DS). Samples were taken at the beginning of the drought stress (t0) and two (t2) and four (t4) weeks after the start of water withholding. Hierarchical clustering is shown for both metabolites (left) and experimental groups (top). The red and blue colors correspond to higher and lower relative metabolite amounts normalized according to *Z*-score.

HCA was also used to assess the pattern of stem metabolites (**Figure 2**). The analysis divided the compounds into two main clusters (1 and 2). Sub-cluster 1a consisted of two flavonoids (luteolin and quercetin-3-*O*-glucoside). Their amount remained constant or increased in the irrigated samples of ‘Leccino’, “GIA DS t4” and “MAU DS t2”. The sub-cluster 1b included two flavonoids (luteolin-7-*O*-glucoside is.2 and apigenin) and two lipophilic compounds (stearic acid and ursolic acid). They were more abundant in the Giarraffa cultivar regardless of time or treatment, but especially in “GIA DS t4” and in “MAU DS t4”. The sub-cluster 2a contained oleuropein, quercetin, and chrysoeriol-7-*O*-glucoside, phenolic compounds whose content decreased in the experimental groups of ‘Giarraffa’ and ‘Leccino’ but increased in the cultivar ‘Maurino’, especially in “MAU DS t2”. The sub-cluster 2b contained 15 lipophilic compounds, with a higher content in the stressed groups of ‘Maurino’ at t2, ‘Giarraffa’ and ‘Leccino’ at t4, as well as “LEC CTRL t4”. Exceptions included neophytadiene and stigmast-5-ene (which decreased significantly in “LEC DS t4”) and LCAlkane 4, which decreased in “MAU DS t2”. The clustering of stem lipophilic and phenolic metabolites was less clear than that of leaf compounds, except for the large clustering of the 15 lipophilic compounds.

The clustering of the experimental group of cultivars showed two distinct clusters. Cluster A was divided into two sub-clusters, the first of which (A1) included ‘Giarraffa’, which had high levels of ursolic acid, luteolin-7-*O*-glucoside is.2 and apigenin. The second sub-cluster (A2) included ‘Maurino’ and ‘Leccino’. Both showed low levels of many fatty acids, including linoleic acid, sugars and other metabolites belonging to sub-cluster 2b. Finally, cluster B included four experimental groups, three of which were exposed to drought stress. Interestingly, the ‘Maurino’ group, after 2 weeks of stress, was grouped together with the stressed ‘Leccino’ and ‘Giarraffa’ groups, stressed by 4 weeks of drought. All of them had higher amounts of the metabolites from sub-cluster 2 and, overall, of some other compounds (such as luteolin, quercetin-3-*O*-glucoside, neophytadiene) belonging to the other sub-clusters of metabolites.

### PCA of leaf metabolite classes

**Figure 3:**
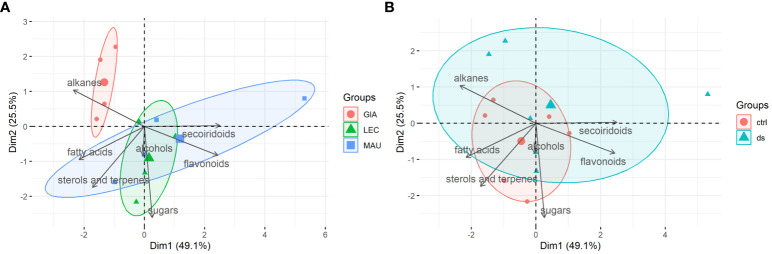
PCA-biplots of principal component 1 (Dim1) and principal component 2 (Dim2) including both observations (experimental groups) and loadings (metabolites classes) of leaf samples. **(A)** Observations were shown as cultivars (GIA, Giarraffa; LEC, Leccino; MAU, Maurino); **(B)** Observations were shown as treatments (ctrl, control; ds, drought stressed).

PCA biplots were used to highlight differences between the experimental groups and identify the classes of leaf metabolites that contributed most to group separation. Principal components 1 (PC1) and 2 (PC2) together accounted for 74.6% of the variation in the leaf data. As shown in **Figure 3A**, all experimental groups of the Giarraffa cultivar were distinct from the other two cultivars and distributed in a restricted area, which corresponded to the class of alkanes. On the other hand, the ‘Leccino’ and ‘Maurino’ groups were less distinct than ‘Giarraffa’ because their distributions partially overlapped. However, ‘Leccino’ was more closely related to the classes of alcohols and sugars, while ‘Maurino’ was best associated with the classes of secoiridoids, sterols and terpenes, and fatty acids. However, when considering the treatment exposure of the experimental groups (**Figure 3B**), the control and stressed samples did not have a clearly defined distribution area. This means that drought stress elicited a diverse range of metabolic responses in the experimental groups, resulting in no distinct or few common metabolic responses to drought stress in the leaves of the three cultivars.

### PCA of stem metabolite classes

**Figure 4:**
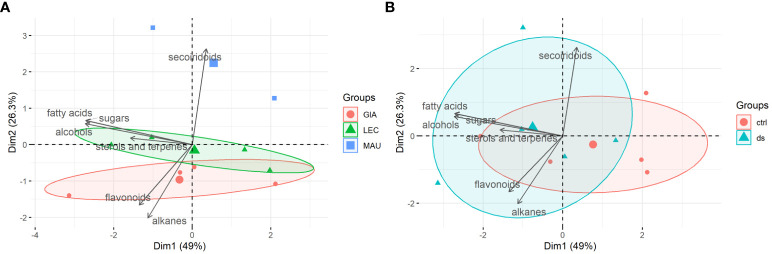
PCA-biplots of principal component 1 (Dim1) and principal component 2 (Dim2) including both observations (experimental groups) and loadings (metabolites classes) of stem samples. **(A)** Observations were shown as cultivars (GIA, Giarraffa; LEC, Leccino; MAU, Maurino); **(B)** Observations were shown as treatments (ctrl, control; ds, drought stressed).

**Figure 4** shows a PCA biplot, a graphical representation of the stem metabolite classes. PC1 and PC2 accounted for a significant 75.3% of the total variation in the stem data, indicating that these two components capture much of the information in the dataset. Like the leaf results in **Figure 3**, the cultivars were separated according to the distribution of stem compounds (**Figure 4A**). This distinction was more pronounced than that observed between the control and drought treatments (**Figure 4B**). PC1 was primarily responsible for determining cultivar distribution. The secoiridoids class was the main contributor to this component, whit flavonoids and alkanes having negative loadings. This suggests that the class of secoiridoids had a significant impact on the distribution of cultivars along PC1. Two cultivars, ‘Leccino’ and ‘Giarraffa’, had similar and partially overlapping distributions. However, ‘Leccino’ was strongly associated with the negative loadings of five lipophilic classes, namely fatty acids, sterols and terpenes, sugars, and alcohols. In contrast, the cultivar Maurino had a different distribution. The distribution area of stem samples from both the stressed and control groups was extensive, with some overlap. This overlap indicated that the stems of the three cultivars did not share a common metabolic response pathway to drought.

## Discussion

In response to drought, phenolic metabolites accumulate primarily in leaves. The metabolomic approach provided information on the quality and quantity of primary and secondary metabolites found in the leaves and stems of three Italian olive cultivars (Giarraffa, Leccino and Maurino) that were either well irrigated or subjected to drought stress for two and four weeks. In this study, we focused on both lipophilic and phenolic compounds; the latter are secondary metabolites known for their antioxidant activity, specifically the ability to act as a defense against reactive oxygen species Morelló et al., 2005. Phenolic compounds were found in all three olive cultivars studied, and most of them increased in response to drought stress only in the leaf. The increase of phenolic compounds in response to drought conditions was demonstrated also in other olive cultivars (Petridis et al., 2012; Talhaoui et al., 2015). Given that phenolic compounds are synthesized through the shikimic acid pathway in photosynthetic cells Del Río, 2003 and that leaves are the primary metabolic source for plants, most of the primary and secondary plant products accumulate in this tissue Ryan et al., 2002. The Spanish olive cultivar ‘Picual’ has also been found to accumulate more phenolic compounds in its leaves than in its stems Del Río, 2003. Groundnut trees exposed to drought accumulated phenolic compounds in both leaves and stems in a genotype-dependent manner; however, the total content and rate of increase were higher in leaves compared to stems Aninbon et al., 2016. Flavonoids are often the most abundant type of phenolic compound identified. They are a large group of phenolic compounds with two benzene rings connected by a three-carbon bridge, which can typically form a third ring Kumar and Pandey, 2013. Flavonoids have traditionally been classified as “effective antioxidants” (orto-dihydroxy B-ring substituted, such as luteolin and quercetin derivatives) and “poor antioxidants” (mono-hydroxy B-ring substituted, such as apigenin derivatives) based on their ability to donate electrons or hydrogen atoms Agati et al., 2012. Flavonoids are particularly known to respond to UV-B stress conditions Ryan et al., 2002; however, they can also accumulate in the leaves of olive trees exposed to drought stress conditions Mechri et al., 2020b. In particular, it was found that the non-toxic and highly soluble flavone-7-glucoside forms of luteolin and apigenin increased under drought stress conditions, whereas the aglycone forms, such as luteolin and apigenin, were found at lower levels under stress conditions compared to the respective controls. As glycosylation replaces the hydroxyl groups of flavonoids, it reduces their antioxidant activity and allows them to accumulate in the vacuole. Flavone glycoconjugates derived from aglycones can accumulate and deplete apigenin and luteolin, respectively. Oleuropein, one of the main phenolic compounds present in olive leaves, is also described to increase under drought conditions, providing more antioxidant protection (Petridis et al., 2012; Talhaoui et al., 2015).

Olive stems have a lower diversity of flavonoids, but they have higher levels of quercetin and luteolin derivatives than leaves, which, due to their high antioxidant capacity, can compensate for the lower flavonoids content of stems. The other type of phenolic compounds detected was the secoiridoids, which are coumarin-like compounds related to the iridoids. Phenolic secoiridoids, like oleuropein, have an oleoside moiety derived from terpene synthesis esterified with a phenolic moiety via a branch of the mevalonic acid pathway Ryan et al., 2002. Ortega-Garcia and Peragòn Ortega-Garcia and Peragòn (2009) identified oleuropein as the main stem compound extracted in the methanol fraction, while Işin et al. (2012) found that oleuropein levels increased in olive leaves with severe water deprivation. In this experiment, we found that oleuropein and oleuropein aglycone were accumulated in response to drought stress, especially in leaf tissue, in contrast to Jimenez-Herrera et al. Jimenez-Herrera et al. (2019), who found that oleuropein was accumulated in olive stem tissue rather than leaf tissue in response to drought.

### Drought stress causes changes in leaf and stem lipophilic metabolites

Like phenolic compounds, leaf tissue contains more primary metabolites than stems. However, higher levels of unsaturated acids, such as alpha-linolenic acid and oleic acid, were observed. Two palmitic acid derivatives were found only in olive stems, which could be related to the lower amount of palmitic acid (a saturated acid) found in the stem than the leaf. In contrast to the phenolic profiles, the treatment significantly altered the profile of primary metabolites in both the stem and the leaf. Drought mainly increased the content of fatty acids (with the exception of linoleic acid), which can act as membrane reinforcement against peroxidation Piccini et al., 2022 or as an energy source for stress recovery Dias et al., 2021. The lower sugar levels available during stress may be due to a reduction in the photosynthetic process or/and an increased use of energy to cope with stress. Furthermore, drought stress decreased sterols content, which may be related to the conversion of sterols into steryl esters, which were linked to membrane reinforcing in drought-tolerant cultivars Rogowska and Szakiel, 2020, or simply an increase in membrane fluidity due to the stress Dias et al., 2021. Unlike previous studies on the UV-B stress response, drought stress did not significantly increase the content of long-chain alkanes. These compounds are involved in cuticle wax thickness, which can be useful in counteracting solar radiation Piccini et al., 2022 and even water loss Bacelar et al., 2004. This was not the case in our study. Sorbitol, a mannitol isomer, is an important osmoprotectant in olive trees. The increase of the levels of polyols, like D-mannitol and galactinol, were reported by Azri et al. (2024) in olive leaves. In the present study, sorbitol did not accumulate during drought stress, but it was found in high concentrations in the leaf, possibly aiding in cell turgor and/or acting as an antioxidant. Because no differences were found, the antioxidant properties of triterpenes and lupeol derivatives were most likely unnecessary after drought stress. The levels of these molecules were also found to be unaffected by stress Dias et al., 2021, with variations occurring only during recovery. Jiménez-Herrera et al. (2019) did not find changes in pentacyclic terpenes (maslinic acid, oleanolic acid, erythrodiol and uvaol) in olive leaves, suggesting that drought does not change the production of these compounds. In contrast, Azri et al. (2024) found a decrease of ursolic acid and oleanolic acid in response to drought.

### Cultivar-specific changes in metabolite profiles in response to drought

Although the data collected allowed for the identification of the most responsive molecules and their different accumulation in the olive leaf and stem, the three cultivars analyzed showed distinct metabolic profiles of drought response. Lipophilic compounds responded more consistently than phenolic compounds within each cultivar, regardless of drought treatment. The two sub-clusters 2b of **Figures 1**, **2** of leaf and stem metabolites, which contain most of the lipophilic compounds, clearly distinguish Giarraffa from the other two cultivars, regardless of treatment. ‘Giarraffa’ leaves contained a high concentration of long chain alkanes, which may be linked to the thickness of the epicuticular wax, allowing this cultivar to avoid excessive water loss. Furthermore, the abundance of palmitic and stearic acids, sterols, and terpenes may enable ‘Giarraffa’ to maintain good membrane fluidity while avoiding excessive permeability (Dias et al., 2021). The potential accumulation of wax on the leaf surface, combined with the physiological responses of ‘Giarraffa’ under drought stress Parri et al., 2023, suggests that this is a typical ‘drought-avoiding’ cultivar Fang and Xiong, 2015. In ‘Giarraffa’, water deficiency reduces stomatal conductance relatively early, resulting in increased stem water content and prolonged soil water availability. Another water-saving strategy of ‘Giarraffa’ is the accumulation of the osmoprotectant D-sorbitol in leaves exposed to two weeks of drought stress. In our previous study, ‘Giarraffa’ had the lowest level of lipid peroxidation and membrane damage, as measured by malondialdehyde content and electrolyte leakage assays Parri et al., 2023. Therefore, there was no need to adjust flavonoid and secoiridoid pools in response to drought, indicating a low level of oxidative stress Agati et al., 2012. However, ‘Giarraffa’ showed a significant decrease in D-glucose, indicating stomatal closure and a lower photosynthetic rate. ‘Giarraffa’ also showed higher levels of alkanes and fatty acids under UV-B conditions, but we did not observe a corresponding accumulation of flavonoids Piccini et al., 2022; this is most likely due to the early physiological response implemented by ‘Giarraffa’ Parri et al., 2023, which allows this cultivar to avoid drought and reduce oxidative stress. In contrast, the metabolic profile of ‘Maurino’ after four weeks of drought stress revealed unusual levels of phenolic and lipophilic compounds, putting it in a separate cluster. As suggested by the high levels of malondialdehyde and electrolyte leakage, the reduction in lipophilic compounds in both leaves and stems could be due to damage on cellular components caused by high levels of oxidative stress Parri et al., 2023. Surprisingly, the stressed group of ‘Maurino’ accumulated flavonoids and secoiridoids, which may aid in the regulation of oxidative stress in both the stem and the leaves. However, unlike *Amaranthus tricolor* Sarker and Oba, 2018 and *Zea mays* Li et al., 2021, which showed higher MDA and EL levels but lower flavonoid content, the antioxidant response of ‘Maurino’ did not allow it to avoid oxidative stress damage. Agati et al. (2012) proposed one possible explanation: flavonoid accumulation as an oxidative stress response occurs particularly in stress-sensitive individuals under severe stress conditions, when the first line of defense against ROS (antioxidant enzymes) is compromised. The metabolite response of ‘Leccino’ is intermediate. Unlike ‘Giarraffa’, ‘Leccino’ contains few long-chain alkanes, sterols, and terpenes and, like ‘Maurino’, the primary fatty acids are oleic and linoleic. However, under drought stress, the phenolic profile changes in a heterogeneous manner. Secoiridoids accumulate only after four weeks of drought, while changes in the flavonoid pool occurred only for a few of them, such as luteolin and apigenin-7-*O*-rutinoside and glucoside, at the cost of a decrease in apigenin and luteolin levels. However, the phenolic profile of this cultivar was associated with lower antioxidant capacity than the other two cultivars under drought stress conditions, as shown by Ferric Ion Reducing Antioxidant Power analysis Cerri et al., 2024.

Finally, analyses of stem and leaf phenolic and lipophilic profiles of the three Italian olive cultivars exposed to drought stress revealed a cultivar-specific response to drought. The cultivars Maurino and Leccino responded more similarly than Giarraffa. However, ‘Maurino’ showed the higher antioxidant response and the greater decrease in most of the lipophilic compounds, indicating a “drought stressed” profile, while ‘Giarraffa’ did not increase flavonoid and secoiridoid pools and showed higher levels of cell wall and cuticle wax components than the other cultivars, supporting the “drought avoidance” pattern shown by the physiological analyses (Parri et al., 2023).

## Data availability statement

The original contributions presented in the study are included in the article/**Supplementary Material**. Further inquiries can be directed to the corresponding author.

## Author contributions

SP: Conceptualization, Formal Analysis, Investigation, Writing – original draft. GC: Conceptualization, Supervision, Writing – review & editing. MR: Conceptualization, Writing – review & editing. CC: Conceptualization, Formal Analysis, Writing – review & editing. DP: Methodology, Resources, Validation, Writing – review & editing. AS: Methodology, Resources, Validation, Writing – review & editing. MD: Formal Analysis, Investigation, Methodology, Resources, Writing – review & editing.
