# Supplementary material for: Comparative metabolomics of leaves and stems of three Italian olive cultivars under drought stress
Source: Front Plant Sci. 2024 Jul 3;15:1408731. doi: 10.3389/fpls.2024.1408731 (PMC11251969; doi:10.3389/fpls.2024.1408731)
Supplement: Supplementary file 1 [file DataSheet_1.pdf]

frontiers

Supplementary Material

List of contents:

Figure S1: scheme of the experimental design (p. 2)

Table S1: phenolic profiles of leaves at T0 (p. 3)

Table S2: phenolic profiles of leaves at T2 (p. 4)

Table S3: phenolic profiles of stems at T0 (p. 5)

Table S4: phenolic profiles of stems at T2 (p. 6)

Table S5: phenolic profiles of stems at T4 (p. 7)

Table S6: lipophilic profiles of leaves at T0 (p. 8)

Table S7: lipophilic profiles of leaves at T2 (p. 9)

Table S8: lipophilic profiles of leaves at T4 (p. 10)

Table S9: lipophilic profiles of stems at T0 (p. 11)

Table S10: lipophilic profiles of stems at T2 (p. 12)

Table S11: lipophilic profiles of stems at T4 (p. 13)

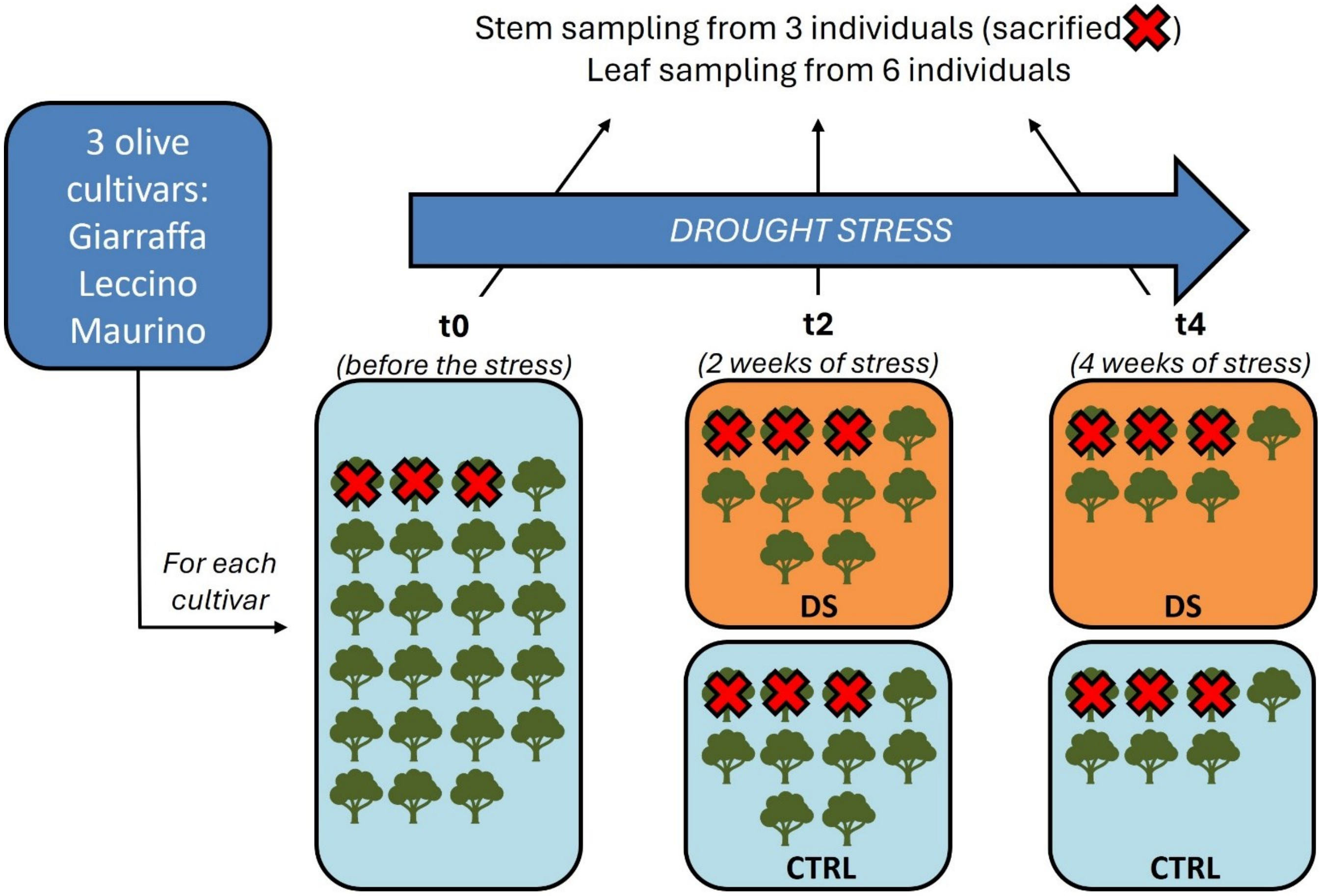

Figure S1. Scheme of the experimental design described in the Material and Methods paragraph.

Table S1. Phenolic profile (mg/g DW) of the **leaves** of the Giarraffa (GIA), Leccino (LEC), and Maurino (MAU) cultivars measured under control (CTRL) conditions and exposed to drought stress (DS) before the start of water deprivation (**T0**). The mean values ± standard deviation (n = 3–4) are presented. Rt—retention time; nd—not detected; is.—isomer.

Rt (min.)	Compound	[M-H] –(m/z)	MS2 (m/z) Fragments	GIA CTRL	GIA DS	LEC CTRL	LEC DS	MAU CTRL	MAU DS
	**secoiridoids**								
10.3	oleuropein aglicone	377	197/153	1.052 ± 0.136	1.026 ± 0.139	nd	nd	0.878 ± 0.011	0.876 ± 0.010
10.8	aldehydic form of decarboxyl elenolic acid	215	197/153/ 171/ 185	0.406 ± 0.073	0.411 ± 0.084	4.498 ± 0.396	4.501 ± 0.404	0.561 ± 0.100	0.559 ± 0.105
14.4	oleuropein	539	377/307/275	1.183 ± 0.207	1.180 ± 0.225	nd	nd	2.469 ± 0.026	2.464 ± 0.023
	**flavonoids**								
11.9	dihydroquercetin	303	285/177/ 125	2.407 ± 0.113	2.412 ± 0.098	2.779 ± 0.074	2.783 ± 0.055	3.263 ± 0.050	3.271 ± 0.033
12.1	luteolin-7-*O*-rutinoside	593	447/285	nd	nd	2.724 ± 0.013	2.722 ± 0.012	3.830 ± 0.144	2.837 ± 0.067
12.1	luteolin-7-*O*-glucoside is.1	447	287/285	2.003 ± 0.030	1.997 ± 0.008	nd	nd	nd	nd
12.4	apigenin-*O*-dideoxyhexoside-hexoxide	449	269	1.812 ± 0.023	1.809 ± 0.018	2.842 ± 0.014	2.842 ± 0.013	2.167 ± 0.002	2.167 ± 0.002
12.8	apigenin-7-*O*-rutinoside is.1	577	269	3.293 ± 0.018	3.296 ± 0.022	5.201 ± 0.073	5.194 ± 0.085	2.570 ± 0.006	2.570 ± 0.007
13.0	apigenin-7-*O*-rutinoside is.2	577	269	2.436 ± 0.183	2.420 ± 0.117	3.085 ± 0.035	3.083 ± 0.036	2.194 ± 0.029	2.198 ± 0.016
13.3	luteolin-7-*O*-glucoside is.2	447	285	3.454 ± 0.090	3.486 ± 0.007	3.017 ± 0.154	3.002 ± 0.058	3.930 ± 0.025	3.932 ± 0.027
13.5	chrysoeriol-7-*O*-glucoside	461	299/446	2.154 ± 0.058	2.151 ± 0.061	nd	nd	2.784 ± 0.025	2.783 ± 0.019
13.9	luteolin-7-*O*-glucoside is.3	447	285	2.158 ± 0.144	2.156 ± 0.108	nd	nd	2.489 ± 0.007	2.488 ± 0.007
15.7	luteolin	285	285	2.515 ± 0.050	2.513 ± 0.025	7.731 ± 0.177	7.737 ± 0.185	4.571 ± 0.007	4.552 ± 0.017
16.7	apigenin-7-*O*-rutinoside	577	269	1.990 ± 0.015	1.994 ± 0.033	2.263 ± 0.014	2.258 ± 0.039	2.145 ± 0.008	2.146 ± 0.009
17.4	apigenin	269	269/225	2.496 ± 0.049	2.497 ± 0.054	6.477 ± 0.099	6.473 ± 0.096	2.720 ± 0.008	2.720 ± 0.007
17.8	diosmetin	299	284	2.526 ± 0.067	2.521 ± 0.054	3.112 ± 0.014	3.112 ± 0.018	3.305 ± 0.014	3.303 ± 0.009

Table S2. Phenolic profile (mg/g DW) of the **leaves** of the Giarraffa (GIA), Leccino (LEC), and Maurino (MAU) cultivars measured under control (CTRL) conditions and exposed to drought stress (DS) for two weeks (**T2**). The mean values ± standard deviation (n = 3–4) are presented. Rt—retention time; nd—not detected; is.—isomer.

**Rt (min.)**	**Compound**	**[M-H] –(m/z)**	**MS2 (m/z) Fragments**	**GIA CTRL**	**GIA DS**	**LEC CTRL**	**LEC DS**	**MAU CTRL**	**MAU DS**
	**secoiridoids**								
10.3	oleuropein aglicone	377	197/153	1.000 ± 0.144	0.811 ± 0.012	nd	nd	0.878 ± 0.011	2.020 ± 0.007
10.8	aldehydic form of decarboxyl elenolic acid	215	197/153/ 171/ 185	0.416 ± 0.095	0.611 ± 0.062	4.505 ± 0.413	0.704 ± 0.049	0.557 ± 0.110	0.801 ± 0.079
14.4	oleuropein	539	377/307/275	1.177 ± 0.247	0.512 ± 0.074	nd	1.208 ± 0.636	2.459 ± 0.027	3.731 ± 0.321
	**flavonoids**								
11.9	dihydroquercetin	303	285/177/ 125	2.418 ± 0.096	2.894 ± 0.060	2.787 ± 0.044	1.920 ± 0.035	3.280 ± 0.017	3.767 ± 0.560
12.1	luteolin-7-*O*-rutinoside	593	447/285	nd	nd	2.720 ± 0.011	1.920 ± 0.070	2.845 ± 0.017	3.417 ± 0.185
12.1	luteolin-7-*O*-glucoside is.1	447	287/285	1.991 ± 0.044	2.302 ± 0.006	nd	nd	nd	nd
12.4	apigenin-*O*-dideoxyhexoside-hexoxide	449	269	1.806 ± 0.021	1.875 ± 0.010	2.842 ± 0.016	2.602 ± 0.058	2.167 ± 0.003	2.228 ± 0.009
12.8	apigenin-7-*O*-rutinoside is.1	577	269	3.299 ± 0.053	3.173 ± 0.042	5.188 ± 0.097	4.438 ± 0.132	2.569 ± 0.008	3.196 ± 0.017
13.0	apigenin-7-*O*-rutinoside is.2	577	269	2.403 ± 0.069	2.311 ± 0.043	3.081 ± 0.038	3.052 ± 0.086	2.202 ± 0.004	2.517 ± 0.049
13.3	luteolin-7-*O*-glucoside is.2	447	285	3.518 ± 0.078	3.725 ± 0.065	2.987 ± 0.252	2.115 ± 0.065	3.933 ± 0.029	5.708 ± 0.030
13.5	chrysoeriol-7-*O*-glucoside	461	299/446	2.147 ± 0.065	2.057 ± 0.039	nd	nd	2.783 ± 0.016	3.447 ± 0.016
13.9	luteolin-7-*O*-glucoside is.3	447	285	2.154 ± 0.126	2.023 ± 0.059	nd	nd	2.488 ± 0.007	2.750 ± 0.036
15.7	luteolin	285	285	2.510 ± 0.049	3.519 ± 0.020	7.743 ± 0.213	3.666 ± 0.096	4.532 ± 0.039	3.458 ± 0.010
16.7	apigenin-7-*O*-rutinoside	577	269	1.999 ± 0.051	1.813 ± 0.040	2.253 ± 0.064	1.802 ± 0.046	2.146 ± 0.010	3.505 ± 1.793
17.4	apigenin	269	269/225	2.497 ± 0.059	2.331 ± 0.060	6.469 ± 0.096	5.102 ± 0.180	2.720 ± 0.007	2.627 ± 0.009
17.8	diosmetin	299	284	2.516 ± 0.042	2.332 ± 0.072	3.111 ± 0.022	2.102 ± 0.041	3.300 ± 0.008	2.579 ± 0.005

Table S3. Phenolic profile (mg/g DW) of the **stems** of the Giarraffa (GIA), Leccino (LEC), and Maurino (MAU) cultivars measured under control (CTRL) conditions and exposed to drought stress (DS) before the start of water deprivation (**T0**). The mean values ± standard deviation (n = 3–4) are presented.

Rt (min.)	Compound	[M-H] –(m/z)	MS2 (m/z) Fragments	GIA CTRL	GIA DS	LEC CTRL	LEC DS	MAU CTRL	MAU DS
	**secoiridoids**								
14.4	oleuropein	539	377/275	0.779 ± 0.125	0.776 ± 0.137	1.410 ± 0.280	1.412 ± 0.284	3.893 ± 0.704	3.891 ± 0.694
15.4	fraxamoside	537	223/375	nd	nd	2.557 ± 0.554	2.560 ± 0.551	nd	nd
	**flavonoids**								
11.9	dihydroquercetin	303	285/177/125	2.992 ± 0.544	2.994 ± 0.541	5.630 ± 1.205	5.615 ± 1.215	3.425 ± 0.592	3.423 ± 0.602
12.8	quercetin-3-*O*-glucoside	463	301	2.336 ± 0.378	2.336 ± 0.379	2.517 ± 0.384	2.518 ± 0.383	2.057 ± 0.226	2.054 ± 0.231
13.5	chrysoeriol-7-*O*-glucoside	461	299/446	1.028 ± 0.134	1.027 ± 0.138	1.159 ± 0.129	1.159 ± 0.129	1.088 ± 0.063	1.092 ± 0.054
13.6	luteolin-7-*O*-glucoside	447	285	9.841 ± 1.608	9.844 ± 1.667	4.284 ± 0.715	4.308 ± 0.740	3.565 ± 0.533	3.566 ± 0.532
15.5	quercetin	301	285/257	0.751 ± 0.036	0.752 ± 0.035	1.272 ± 0.103	1.272 ± 0.107	1.049 ± 0.052	1.050 ± 0.052
17.4	apigenin	269	149	2.134 ± 0.293	2.131 ± 0.287	1.195 ± 0.090	1.195 ± 0.088	0.944 ± 0.028	0.944 ± 0.030
15.8	luteolin	285	285	1.109 ± 0.122	1.106 ± 0.110	1.199 ± 0.076	1.198 ± 0.086	1.152 ± 0.062	1.151 ± 0.066

Table S4. Phenolic profile (mg/g DW) of the **stems** of the Giarraffa (GIA), Leccino (LEC), and Maurino (MAU) cultivars measured under control (CTRL) conditions and exposed to drought stress (DS) for two weeks (**T2**). The mean values ± standard deviation (n = 3–4) are presented. Rt—retention time; nd—not detected.

Rt (min.)	Compound	[M-H] –(m/z)	MS2 (m/z) Fragments	GIA CTRL	GIA DS	LEC CTRL	LEC DS	MAU CTRL	MAU DS
	**secoiridoids**								
14.4	oleuropein	539	377/275	0.773 ± 0.149	0.687 ± 0.171	1.141 ± 0.289	1.306 ± 0.242	3.889 ± 0.684	4.382 ± 0.870
15.4	fraxamoside	537	223/375	nd	nd	2.562 ± 0.549	2.646 ± 0.553	nd	nd
	**flavonoids**								
11.9	dihydroquercetin	303	285/177/ 125	2.997 ± 0.538	2.718 ± 0.625	5.600 ± 1.225	4.025 ± 0.841	3.421 ± 0.612	4.036 ± 0.735
12.8	quercetin-3-*O*-glucoside	463	301	2.336 ± 0.381	2.120 ± 0.444	2.519 ± 0.381	2.036 ± 0.251	2.054 ± 0.230	2.591 ± 0.324
13.5	chrysoeriol-7-*O*-glucoside	461	299/446	1.025 ± 0.142	0.961 ± 0.164	1.159 ± 0.130	0.866 ± 0.088	1.097 ± 0.058	1.406 ± 0.112
13.6	luteolin-7-*O*-glucoside	447	285	9.848 ± 1.726	8.711 ± 2.143	4.332 ± 0.765	2.662 ± 0.410	3.568 ± 0.532	4.287 ± 0.623
15.5	quercetin	301	285/257	0.753 ± 0.035	0.723 ± 0.042	1.271 ± 0.111	0.969 ± 0.094	1.050 ± 0.052	1.326 ± 0.124
17.4	apigenin	269	149	2.128 ± 0.281	1.935 ± 0.336	1.196 ± 0.086	0.908 ± 0.059	0.945 ± 0.033	1.557 ± 0.253
15.8	luteolin	285	285	1.102 ± 0.102	1.032 ± 0.118	1.197 ± 0.095	0.857 ± 0.051	1.150 ± 0.069	1.818 ± 0.178

Table S5. Phenolic profile (mg/g DW) of the **stems** of the Giarraffa (GIA), Leccino (LEC), and Maurino (MAU) cultivars measured under control (CTRL) conditions and exposed to drought stress (DS) for four weeks (**T4**). The mean values ± standard deviation (n = 3–4) are presented. Rt—retention time; nd—not detected.

**Rt (min.)**	**Compound**	**[M-H] –(m/z)**	**MS2 (m/z) Fragments**	**GIA CTRL**	**GIA DS**	**LEC CTRL**	**LEC DS**
	**secoiridoids**						
14.4	oleuropein	539	377/275	0.723 ± 0.096	0.924 ± 0.131	1.238 ± 0.232	1.333 ± 0.294
15.4	fraxamoside	537	223/375	nd	nd	2.191 ± 0.396	0.979 ± 0.245
	**flavonoids**						
11.9	dihydroquercetin	303	285/177/ 125	2.823 ± 0.373	3.914 ± 0.483	4.854 ± 0.931	3.757 ± 0.773
12.8	quercetin-3-*O*-glucoside	463	301	2.213 ± 0.260	2.606 ± 0.308	2.158 ± 0.276	2.230 ± 0.426
13.5	chrysoeriol-7-*O*-glucoside	461	299/446	0.996 ± 0.107	1.249 ± 0.078	1.005 ± 0.119	1.122 ± 0.258
13.6	luteolin-7-*O*-glucoside	447	285	9.301 ± 1.178	13.66 ± 2.081	3.751 ± 0.583	4.407 ± 0.857
15.5	quercetin	301	285/257	0.762 ± 0.024	0.946 ± 0.048	1.106 ± 0.083	0.866 ± 0.052
17.4	apigenin	269	149	2.050 ± 0.187	2.091 ± 0.230	1.019 ± 0.064	1.147 ± 0.101
15.8	luteolin	285	285	1.100 ± 0.070	1.376 ± 0.117	1.029 ± 0.068	0.976 ± 0.069

Table S6. Lipophilic profile (mg/g DW) of the **leaves** of the Giarraffa (GIA), Leccino (LEC), and Maurino (MAU) cultivars measured under control (CTRL) conditions and exposed to drought stress (DS) before the start of water deprivation (**T0**). The mean values ± standard deviation (n = 3–4) are presented. Rt—retention time; nd—not detected.

Rt (min.)	Compound	GIA CTRL	GIA DS	LEC CTRL	LEC DS	MAU CTRL	MAU DS
	**sterols and terpenes**						
34.0	neophytadiene	0.966 ± 0.019	0.963 ± 0.014	0.608 ± 0.011	0.611 ± 0.008	0.647 ± 0.014	0.648 ± 0.014
42.1	phytol	nd	nd	0.588 ± 0.002	0.589 ± 0.001	nd	nd
55.4	squalene	nd	nd	0.613 ± 0.011	0.608 ± 0.004	0.601 ± 0.011	0.605 ± 0.007
68.0	β - amyrin	1.321 ± 0.102	1.324 ± 0.212	0.689 ± 0.010	0.689 ± 0.013	0.731 ± 0.0315	0.733 ± 0.038
69.0	α - amyrin	1.419 ± 0.419	1.442 ± 0.408	0.647 ± 0.006	0.646 ± 0.000	0.677 ± 0.011	0.675 ± 0.013
72.8	lupeol derivatives	2.311 ± 0.534	2.307 ± 0.547	0.679 ± 0.010	0.679 ± 0.001	0.654 ± 0.011	0.655 ± 0.011
73.4	ursolic acid	1.689 ± 0.593	1.682 ± 0.547	0.831 ± 0.027	0.830 ± 0.023	1.085 ± 0.107	1.083 ± 0.120
73.6	ursolic acid aldeyde	nd	nd	0.672 ± 0.018	0.668 ± 0.036	0.731 ± 0.029	0.730 ± 0.036
	**sugars**						
35.5	α - D - mannopyranose	nd	nd	0.173 ± 0.006	0.173 ± 0.002	0.196 ± 0.010	0.194 ± 0.010
37.7	D - glucose	0.250 ± 0.009	0.251 ± 0.008	0.188 ± 0.003	0.188 ± 0.002		
	**alcohols**						
36.3	D - sorbitol	1.092 ± 0.034	1.094 ± 0.039	0.814 ± 0.010	0.815 ± 0.013	0.875 ± 0.094	0.871 ± 0.007
63.0	α - tocopherol	nd	nd	0.670 ± 0.010	0.675 ± 0.011	nd	nd
	**fatty acids**						
39.2	palmitic acid	11.393 ± 0.318	11.392 ± 0.298	7.411 ± 0.087	7.416 ± 0.150	7.750 ± 0.668	7.752 ± 0.535
42.8	linoleic acid	nd	nd	6.472 ± 0.030	6.468 ± 0.010	6.419 ± 0.008	6.420 ± 0.003
42.9	α - linolenic acid	10.450 ± 0.019	10.497 ± 0.043	nd	nd	nd	nd
43.1	oleic acid	nd	nd	7.372 ± 0.415	7.374 ± 0.548	6.635 ± 0.005	6.635 ± 0.023
43.2	oleic acid derivative	nd	nd	6.505 ± 0.057	6.508 ± 0.0778	6.417 ± 0.006	6.417 ± 0.004
43.7	stearic acid	10.865 ± 0.057	10.867 ± 0.072	7.011 ± 0.109	6.989 ± 0.090	7.739 ± 0.100	7.236 ± 0.185
45.5	α - monopalmitin	10.283 ± 0.031	10.283 ± 0.036	6.469 ± 0.036	6.464 ± 0.014	6.544 ± 0.033	6.549 ± 0.041
50.7	α - monopalmitin derivative	10.420 ± 0.028	10.420 ± 0.050	6.540 ± 0.010	6.537 ± 0.009	6.701 ± 0.098	6.704 ± 0.096
	**alkanes**						
57.6	long chain alkane 1	1.603 ± 0.097	1.606 ± 0.107	0.897 ± 0.012	0.896 ± 0.003	1.122 ± 0.088	1.117 ± 0.104
62.6	long chain alkane 2	2.109 ± 0.219	2.107 ± 0.226	1.193 ± 0.050	1.191 ± 0.047	1.544 ± 0.138	1.545 ± 0.169
67.7	long chain alkane 3	2.931 ± 0.737	2.929 ± 0.760	1.630 ± 0.141	1.625 ± 0.062	2.289 ± 0.343	2.286 ± 0.208
73.0	long chain alkane 4	1.993 ± 0.448	1.999 ± 0.189	1.004 ± 0.021	1.003 ± 0.076	1.230 ± 0.111	1.229 ± 0.065

Table S7. Lipophilic profile (mg/g DW) of the leaves of the Giarraffa (GIA), Leccino (LEC), and Maurino (MAU) cultivars measured under control (CTRL) conditions and exposed to drought stress (DS) for two weeks (**T2**). The mean values ± standard deviation (n = 3–4) are presented. Rt—retention time; nd—not detected.

Rt (min.)	Compound	GIA CTRL	GIA DS	LEC CTRL	LEC DS	MAU CTRL	MAU DS
	**sterols and terpenes**						
34.0	neophytadiene	0.961 ± 0.012	nd	0.613 ± 0.006	0.604 ± 0.011	0.649 ± 0.027	0.772 ± 0.002
42.1	phytol	nd	nd	0.590 ± 0.001	0.571 ± 0.002	0.597 ± 0.012	nd
55.4	squalene	nd	nd	0.604 ± 0.008	0.588 ± 0.007	0.610 ± 0.004	0.798 ± 0.005
68.0	β - amyrin	1.327 ± 0.367	1.187 ± 0.045	0.689 ± 0.017	0.688 ± 0.081	0.735 ± 0.044	0.905 ± 0.030
69.0	α - amyrin	1.425 ± 0.399	1.149 ± 0.127	0.645 ± 0.006	0.678 ± 0.050	0.674 ± 0.014	0.946 ± 0.050
72.8	lupeol derivatives	2.302 ± 0.686	1.523 ± 0.092	0.678 ± 0.010	0.785 ± 0.057	0.658 ± 0.016	1.246 ± 0.045
73.4	ursolic acid	1.675 ± 0.511	1.474 ± 0.006	0.829 ± 0.049	1.098 ± 0.133	1.082 ± 0.136	1.114 ± 0.043
73.6	ursolic acid aldeyde	1.348 ± 0.570	nd	0.662 ± 0.017	0.732 ± 0.063	0.128 ± 0.048	0.883 ± 0.026
	**sugars**						
35.5	α - D - mannopyranose	nd	nd	0.173 ± 0.002	0.165 ± 0.005	0.192 ± 0.022	0.192 ± 0.002
37.7	D - glucose	0.252 ± 0.008	nd	0.188 ± 0.004	0.179 ± 0.008	0.215 ± 0.026	0.197 ± 0.002
	**alcohols**						
36.3	D - sorbitol	1.095 ± 0.045	nd	0.817 ± 0.022	0.884 ± 0.055	0.868 ± 0.108	0.861 ± 0.018
63.0	α - tocopherol	nd	nd	0.680 ± 0.013	0.731 ± 0.074	0.669 ± 0.017	nd
	**fatty acids**						
39.2	palmitic acid	11.39 ± 0.28	11.47 ± 0.09	7.422 ± 0.218	7.203 ± 0.149	7.754 ± 0.421	12.26 ± 5.20
42.8	linoleic acid	nd	nd	6.466 ± 0.018	6.135 ± 0.005	6.422 ± 0.010	8.323 ± 0.004
42.9	α - linolenic acid	10.50 ± 0.10	10.74 ± 0.02	6.384 ± 0.034	nd	nd	nd
43.1	oleic acid	nd	nd	7.376 ± 0.681	6.611 ± 0.021	6.635 ± 0.045	9.070 ± 1.052
43.2	oleic acid derivative	nd	nd	6.511 ± 0.148	6.177 ± 0.016	6.417 ± 0.003	8.333 ± 0.015
43.7	stearic acid	10.84 ± 0.139	11.14 ± 0.071	6.967 ± 0.129	6.798 ± 0.077	7.233 ± 0.269	8.904 ± 0.043
45.5	α - monopalmitin	10.28 ± 0.042	10.66 ± 0.015	6.459 ± 0.009	6.229 ± 0.027	6.554 ± 0.063	8.415 ± 0.015
50.7	α - monopalmitin derivative	10.42 ± 0.072	10.76 ± 0.041	6.533 ± 0.018	6.350 ± 0.046	6.707 ± 0.112	8.470 ± 0.132
	**alkanes**						
57.6	long chain alkane 1	1.610 ± 0.118	1.594 ± 0.066	0.894 ± 0.0139	0.926 ± 0.042	1.113 ± 0.126	1.236 ± 0.023
62.6	long chain alkane 2	2.105 ± 0.234	2.233 ± 0.080	1.190 ± 0.089	1.297 ± 0.113	1.545 ± 0.230	1.552 ± 0.057
67.7	long chain alkane 3	2.926 ± 0.806	2.656 ± 0.0725	1.620 ± 0.195	2.127 ± 0.277	2.283 ± 0.490	1.908 ± 0.135
73.0	long chain alkane 4	2.001 ± 0.477	1.760 ± 0.046	1.002 ± 0.137	1.364 ± 0.179	1.229 ± 0.133	1.255 ± 0.017

Table S8. Lipophilic profile (mg/g DW) of the leaves of the Giarraffa (GIA), Leccino (LEC), and Maurino (MAU) cultivars measured under control (CTRL) conditions and exposed to drought stress (DS) for four weeks (**T4**). The mean values ± standard deviation (n = 3–4) are presented. Rt—retention time; nd—not detected.

**Rt (min.)**	**Compound**	**GIA CTRL**	**GIA DS**	**LEC CTRL**	**LEC DS**	**MAU CTRL**	**MAU DS**
	**sterols and terpenes**						
34.0	neophytadiene	1.034 ± 0.002	nd	0.778 ± 0.007	nd	1.066 ± 0.221	0.361 ± 0.002
42.1	phytol	nd	nd	0.753 ± 0.003	nd	nd	0.359 ± 0.004
55.4	squalene	nd	nd	0.604 ± 0.008	0.588 ± 0.007	0.610 ± 0.004	0.798 ± 0.005
68.0	β - amyrin	1.327 ± 0.367	1.187 ± 0.045	0.689 ± 0.017	0.688 ± 0.081	0.735 ± 0.044	0.905 ± 0.030
69.0	α - amyrin	nd	1.358 ± 0.613	0.841 ± 0.022	0.905 ± 0.010	0.996 ± 0.028	0.417 ± 0.002
72.8	lupeol derivatives	1.579 ± 0.355	nd	1.026 ± 0.065	nd	1.294 ± 0.071	0.547 ± 0.040
73.4	ursolic acid	1.922 ± 0.587	1.397 ± 0.219	1.169 ± 0.086	1.228 ± 0.331	1.261 ± 0.083	0.628 ± 0.058
73.6	ursolic acid aldeyde	nd	nd	0.861 ± 0.037	0.950 ± 0.060	1.000 ± 0.065	0.380 ± 0.008
	**sugars**						
35.5	α - D - mannopyranose	0.276 ± 0.003	nd	0.224 ± 0.004	0.217 ± 0.003	0.246 ± 0.013	0.122 ± 0.008
37.7	D - glucose	0.290 ± 0.003	nd	0.428 ± 0.326	0.225 ± 0.007	0.263 ± 0.018	0.131 ± 0.009
	**alcohols**						
36.3	D - sorbitol	1.327 ± 0.023	1.073 ± 0.010	1.172 ± 0.050	1.287 ± 0.126	1.106 ± 0.090	1.282 ± 0.173
63.0	α - tocopherol	nd	1.343 ± 0.353	0.813 ± 0.022	0.841 ± 0.175	nd	0.447 ± 0.019
	**fatty acids**						
39.2	palmitic acid	12.10 ± 0.204	11.20 ± 0.118	9.179 ± 0.093	10.08 ± 0.359	10.72 ± 0.347	4.524 ± 0.113
42.8	linoleic acid	10.99 ± 0.003	10.43 ± 0.004	8.189 ± 0.005	9.237 ± 0.238	9.624 ± 0.012	3.899 ± 0.004
42.9	α - linolenic acid	nd	10.64 ± 0.028	nd	nd	nd	4.001 ± 0.007
43.1	oleic acid	11.21 ± 0.047	nd	8.599 ± 0.030	10.19 ± 1.359	9.793 ± 0.053	nd
43.2	oleic acid derivative	nd	nd	8.268 ± 0.006	9.191 ± 0.032	9.625 ± 0.003	3.892 ± 0.004
43.7	stearic acid	11.67 ± 0.132	10.91 ± 0.082	8.797 ± 0.062	9.589 ± 0.067	10.29 ± 0.217	4.287 ± 0.070
45.5	α - monopalmitin	11.09 ± 0.027	10.48 ± 0.012	8.273 ± 0.012	9.169 ± 0.014	9.720 ± 0.046	3.950 ± 0.014
50.7	α - monopalmitin derivative	11.25 ± 0.048	10.61 ± 0.028	8.405 ± 0.029	9.278 ± 0.032	9.832 ± 0.086	3.999 ± 0.023
	**alkanes**						
57.6	long chain alkane 1	1.684 ± 0.021	1.589 ± 0.125	1.068 ± 0.014	1.176 ± 0.078	1.389 ± 0.103	0.592 ± 0.029
62.6	long chain alkane 2	2.297 ± 0.158	2.360 ± 0.435	1.448 ± 0.076	1.413 ± 0.242	1.740 ± 0.188	0.731 ± 0.057
67.7	long chain alkane 3	3.245 ± 0.398	2.871 ± 0.742	1.992 ± 0.133	1.860 ± 0.268	2.149 ± 0.356	0.903 ± 0.089
73.0	long chain alkane 4	2.215 ± 0.505	2.371 ± 1.068	1.451 ± 0.118	1.388 ± 0.248	1.381 ± 0.248	0.538 ± 0.026

Table S9. Lipophilic profile (mg/g DW) of the **stems** of the Giarraffa (GIA), Leccino (LEC), and Maurino (MAU) cultivars measured under control (CTRL) conditions and exposed to drought stress (DS) before the start of water deprivation (**T0**). The mean values ± standard deviation (n = 3–4) are presented. Rt—retention time; nd—not detected.

Rt (min.)	Compound	GIA CTRL	GIA DS	LEC CTRL	LEC DS	MAU CTRL	MAU DS
	**sterols and terpenes**						
67.4	stigmast-5-ene	0.731 ± 0.002	0.730 ± 0.003	0.720 ± 0.009	0.720 ± 0.008	0.726 ± 0.012	0.727 ± 0.013
71.7	lupeol derivatives	0.581 ± 0.003	0.582 ± 0.001	0.370 ± 0.026	0.0365 ± 0.027	0.356 ± 0.005	0.356 ± 0.003
73.0	ursolic acid	0.647 ± 0.006	0.647 ± 0.003	0.490 ± 0.010	0.490 ± 0.007	0.604 ± 0.006	0.603 ± 0.005
73.2	ursolic acid aldeyde	0.595 ± 0.005	0.593 ± 0.002	0.354 ± 0.006	0.353 ± 0.005	0.356 ± 0.006	0.356 ± 0.003
	**sugars**						
35.5	α - D - mannopyranose	0.139 ± 0.001	0.139 ± 0.001	0.097 ± 0.006	0.091 ± 0.002	0.088 ± 0.001	0.087 ± 0.000
37.5	D - glucose	0.145 ± 0.005	0.143 ± 0.003	0.087 ± 0.006	0.088 ± 0.003	0.087 ± 0.001	0.087 ± 0.001
51.4	turanose	0.147 ± 0.006	0.145 ± 0.003	0.096 ± 0.013	0.092 ± 0.006	0.349 ± 0.453	0.218 ± 0.226
	**alcohols**						
36.2	D - sorbitol	0.663 ± 0.006	0.662 ± 0.005	0.782 ± 0.031	0.784 ± 0.024	0.354 ± 0.005	0.353 ± 0.003
44.8	pentadecan-1-ol derivative	0.563 ± 0.006	0.562 ± 0.002	0.391 ± 0.000	0.389 ± 0.003	0.355 ± 0.005	0.354 ± 0.002
	**fatty acids**						
39.1	palmitic acid	6.475 ± 0.005	6.475 ± 0.002	4.693 ± 0.006	4.691 ± 0.009	5.440 ± 0.013	5.440 ± 0.023
42.7	α - linolenic acid	6.063 ± 0.005	6.064 ± 0.003	3.535 ± 0.006	3.533 ± 0.001	3.973 ± 0.013	3.971 ± 0.017
42.9	oleic acid	6.109 ± 0.003	6.109 ± 0.003	3.621 ± 0.009	3.620 ± 0.005	4.016 ± 0.012	4.013 ± 0.010
43.5	stearic acid	6.303 ± 0.005	6.306 ± 0.004	4.244 ± 0.041	4.243 ± 0.032	4.816 ± 0.006	4.814 ± 0.005
45.3	α - monopalmitin	6.107 ± 0.005	6.105 ± 0.003	3.631 ± 0.001	3.632 ± 0.001	4.066 ± 0.005	4.066 ± 0.005
45.6	palmitic acid derivative 1	6.207 ± 0.005	6.207 ± 0.003	3.854 ± 0.011	3.851 ± 0.004	4.325 ± 0.025	4.325 ± 0.026
49.3	palmitic acid derivative 2	6.116 ± 0.006	6.117 ± 0.003	3.513 ± 0.006	3.514 ± 0.005	4.090 ± 0.010	4.089 ± 0.010
50.5	α – monopalmitin derivative	6.724 ± 0.012	6.724 ± 0.003	4.410 ± 0.000	4.415 ± 0.021	6.093 ± 0.030	6.089 ± 0.035
54.5	monostearin	6.759 ± 0.010	6.756 ± 0.007	4.447 ± 0.015	4.442 ± 0.019	6.163 ± 0.082	6.168 ± 0.050

Table S10. Lipophilic profile (mg/g DW) of the **stems** of the Giarraffa, Leccino, and Maurino cultivars measured under control (CTRL) conditions and exposed to drought stress (DS) for two weeks (**T2**). The mean values ± standard deviation (n = 3–4) are presented. Rt—retention time; nd—not detected.

Rt (min.)	Compound	GIA CTRL	GIA DS	LEC CTRL	LEC DS	MAU CTRL	MAU DS
	**sterols and terpenes**						
67.4	stigmast-5-ene	0.729 ± 0.004	0.733 ± 0.007	0.719 ± 0.011	0.720 ± 0.008	0.727 ± 0.016	0.940 ± 0.034
71.7	lupeol derivatives	0.582 ± 0.033	0.522 ± 0.000	0.361 ± 0.041	0.494 ± 0.008	0.355 ± 0.001	0.696 ± 0.002
73.0	ursolic acid	0.647 ± 0.020	0.642 ± 0.035	0.490 ± 0.011	0.596 ± 0.020	0.601 ± 0.016	nd
73.2	ursolic acid aldeyde	0.592 ± 0.022	0.612 ± 0.027	0.352 ± 0.005	0.481 ± 0.022	0.355 ± 0.002	0.696 ± 0.004
	**sugars**						
35.5	α - D - mannopyranose	0.139 ± 0.004	0.132 ± 0.000	0.085 ± 0.002	nd	0.087 ± 0.000	0.171 ± 0.000
37.5	D - glucose	0.141 ± 0.003	0.135 ± 0.000	0.089 ± 0.000	0.107 ± 0.000	0.087 ± 0.000	0.171 ± 0.000
51.4	turanose	0.142 ± 0.004	0.135 ± 5.321	0.087 ± 0.000	nd	0.087 ± 0.000	0.171 ± 0.00
	**alcohols**						
36.2	D - sorbitol	0.660 ± 0.024	0.705 ± 0.002	0.786 ± 0.023	0.566 ± 0.005	0.353 ± 0.000	0.692 ± 0.000
44.8	pentadecan-1-ol derivative	0.560 ± 0.008	0.546 ± 0.003	0.387 ± 0.006	0.489 ± 0.011	0.353 ± 0.000	0.691 ± 0.000
	**fatty acids**						
39.1	palmitic acid	6.476 ± 0.094	6.304 ± 0.002	4.688 ± 0.014	5.531 ± 0.015	5.439 ± 0.033	8.191 ± 0.039
42.7	α - linolenic acid	6.064 ± 0.171	5.754 ± 0.007	3.531 ± 0.006	4.743 ± 0.006	3.970 ± 0.024	7.639 ± 0.001
42.9	oleic acid	6.110 ± 0.185	5.775 ± 0.006	3.619 ± 0.001	4.823 ± 0.007	4.010 ± 0.012	7.639 ± 0.001
43.5	stearic acid	6.309 ± 0.093	6.141 ± 0.003	4.242 ± 0.029	5.170 ± 0.008	4.811 ± 0.007	8.024 ± 0.030
45.3	α - monopalmitin	6.103 ± 0.154	5.822 ± 0.001	3.634 ± 0.002	4.786 ± 0.003	4.066 ± 0.013	7.639 ± 0.001
45.6	palmitic acid derivative 1	6.206 ± 0.144	5.945 ± 0.002	3.848 ± 0.006	4.935 ± 0.006	4.325 ± 0.028	7.891 ± 0.035
49.3	palmitic acid derivative 2	6.117 ± 0.154	5.834 ± 0.004	3.515 ± 0.013	4.790 ± 0.005	4.089 ± 0.011	7.639 ± 0.001
50.5	α – monopalmitin derivative	6.724 ± 0.190	6.380 ± 0.007	4.420 ± 0.043	5.268 ± 0.003	6.085 ± 0.078	8.378 ± 0.026
54.5	monostearin	6.753 ± 0.204	6.381 ± 0.005	4.438 ± 0.038	5.290 ± 0.015	6.174 ± 0.082	8.364 ± 0.083

Table S11. Lipophilic profile (mg/g DW) of the **stems** of the Giarraffa (GIA), Leccino (LEC), and Maurino (MAU) cultivars measured under control (CTRL) conditions and exposed to drought stress (DS) for four weeks (**T4**). The mean values ± standard deviation (n = 3–4) are presented. Rt—retention time; nd—not detected.

**Rt (min.)**	**Compound**	**GIA CTRL**	**GIA DS**	**LEC CTRL**	**LEC DS**
	**sterols and terpenes**				
67.4	stigmast-5-ene	0.635 ± 0.035	0.932 ± 0.028	0.882 ± 0.093	0.901 ± 0.006
71.7	lupeol derivatives	0.418 ± 0.054	0.876 ± 0.061	0.756 ± 0.000	nd
73.0	ursolic acid	0.510 ± 0.056	0.970 ± 0.049	0.870 ± 0.083	0.807 ± 0.005
73.2	ursolic acid aldeyde	0.393 ± 0.045	0.880 ± 0.089	nd	nd
	**sugars**				
35.5	α - D - mannopyranose	0.086 ± 0.001	0.184 ± 0.001	0.190 ± 0.004	nd
37.5	D - glucose	0.091 ± 0.000	0.188 ± 0.001	0.193 ± 0.006	0.178 ± 0.000
51.4	turanose	0.087 ± 0.002	0.184 ± 0.001	0.194 ± 0.007	nd
	**alcohols**				
36.2	D - sorbitol	0.511 ± 0.003	0.954 ± 0.008	0.990 ± 0.177	0.776 ± 0.004
44.8	pentadecan-1-ol derivative	0.360 ± 0.002	0.730 ± 0.015	nd	0.732 ± 0.006
	**fatty acids**				
39.1	palmitic acid	4.419 ± 0.004	8.373 ± 0.008	8.635 ± 0.254	8.341 ± 0.009
42.7	α - linolenic acid	3.616 ± 0.002	8.019 ± 0.005	8.364 ± 0.055	7.877 ± 0.002
42.9	oleic acid	4.098 ± 0.004	8.252 ± 0.009	8.541 ± 0.184	8.192 ± 0.004
43.5	stearic acid	3.638 ± 0.002	8.041 ± 0.003	8.350 ± 0.043	7.921 ± 0.002
45.3	α - monopalmitin	3.803 ± 0.002	8.132 ± 0.000	8.419 ± 0.094	8.026 ± 0.003
45.6	palmitic acid derivative 1	3.653 ± 0.001	8.056 ± 0.001	8.363 ± 0.053	7.933 ± 0.002
49.3	palmitic acid derivative 2	4.371 ± 0.007	8.715 ± 0.004	8.865 ± 0.425	8.661 ± 0.008
50.5	α – monopalmitin derivative	4.406 ± 0.009	8.751 ± 0.011	8.862 ± 0.422	8.674 ± 0.006
